# Community, Time, and (Con)text: A Dynamical Systems Analysis of Online Communication and Community Health among Open‐Source Software Communities

**DOI:** 10.1111/cogs.13134

**Published:** 2022-05-17

**Authors:** Alexandra Paxton, Nelle Varoquaux, Chris Holdgraf, R. Stuart Geiger

**Affiliations:** ^1^ Department of Psychological Sciences University of Connecticut; ^2^ Center for the Ecological Study of Perception and Action University of Connecticut; ^3^ TIMC, Univ. Grenoble Alpes, CNRS, Grenoble INP; ^4^ 2i2c Code for Science and Society; ^5^ Department of Statistics University of California, Berkeley; ^6^ Department of Communication University of California, San Diego; ^7^ Halıcıoğlu Data Science Institute University of California, San Diego

**Keywords:** Community dynamics, Natural language processing, Open‐source software, Dynamical systems, Volunteering

## Abstract

Free and open‐source software projects have become essential digital infrastructure over the past decade. These projects are largely created and maintained by unpaid volunteers, presenting a potential vulnerability if the projects cannot recruit and retain new volunteers. At the same time, their development on open collaborative development platforms provides a nearly complete record of the community's interactions; this affords the opportunity to study naturally occurring language dynamics at scale and in a context with massive real‐world impact. The present work takes a dynamical systems view of language to understand the ways in which communicative context and community membership shape the emergence and impact of language use—specifically, sentiment and expressions of gratitude. We then present evidence that these language dynamics shape newcomers' likelihood of returning, although the specific impacts of different community responses are crucially modulated by the context of the newcomer's first contact with the community.

## 1 Introduction

With access to the Internet and programming skills on the rise, more people than ever are contributing to and relying on *free and open‐source software* (FOSS). The defining characteristics of this special class of software projects have changed somewhat over the past several decades (e.g., Crowston & Howison, 2005; Holtgrewe & Werle, 2001). FOSS projects are often defined by their release under various licenses that allow anyone to use, change, and share the software. Many FOSS projects also follow a model of “commons‐based peer production” (Benkler & Nissenbaum, 2006) based on open contributions, similar to Wikipedia. Despite the variety in their policies and practices, FOSS projects are widely known for their impressive networks of volunteer maintainers and developers, with a recent survey finding that half of FOSS contributors are unpaid for their work on the projects (Nagle et al., 2020).

FOSS has become a critical digital infrastructure across domains and around the world (Eghbal, 2016), despite some critics' concerns about possible risks (Goode, 2005; Hauge, Ayala, & Conradi, 2010; Silic & Back, 2016). Their reliance on volunteer labor stands as one of their most critical—and, potentially, most preventable—sources of potential failure (cf. Eghbal, 2016; Norton, 2018). The continued security, maintenance, and development of FOSS projects hinge on sustaining the health of their community—that is, the recruitment of new contributors and the retention of existing contributors.

With this application in mind, the current project uses a real‐world context to understand the impact of different patterns of communication within communities. While a person's decision to enter or leave a FOSS project is no doubt affected by a range of factors (e.g., financial concerns, availability of free time), the impact of communication within communities has been identified by previous research as a critical potential driver of that engagement or disengagement (e.g., de Magalhães, 2017). Long‐time contributors to FOSS projects may meet one another in person (e.g., at sprints or conferences), but most newcomers or potential contributors to a project only have computer‐mediated text‐based interactions with other members of the community (e.g., Dabbish, Stuart, Tsay, & Herbsleb, 2012). The current work examines the traces of these digital interactions to identify patterns of community dynamics and the impacts of those patterns on the community's ability to retain newcomers.

We also use this project as an opportunity to examine community dynamics in real‐world, soft‐assembled, self‐motivated groups. Open online communities provide unique ecological opportunities to study social dynamics at multiple levels (e.g., Hill & Shaw, 2019; Schroeder & Taylor, 2015). Because these communities collaborate primarily through GitHub (although not exclusively; cf. Yasir, Michael, Savarimuthu, & Licorish, 2018), this study allows us to examine a nearly complete historical record of a community's interactions. As such, we are able to test predictions of community‐specific language structures in a naturalistic setting (Rączaszek‐Leonardi & Kelso, 2008).

### 1.1 Ethnographic and qualitative studies of software engineers

Ethnographers have qualitatively studied software development and FOSS communities for decades (Coleman & Hill, 2005; Kelty, 2005). We turn our attention to a more recent development: the dominance of GitHub, a “social coding” platform that facilitates programming *and* coordination. Previous qualitative and ethnographic work has conducted deep dives into the motivations and experiences both of software developers generally and of software developers working specifically on this platform. We review these findings here as a basis for understanding the motivation and culture of FOSS communities broadly.

#### 1.1.1 FOSS projects as communities

We use the term “community” to describe FOSS projects. Admittedly, this is a term used in quite different ways by both researchers and practitioners. For some, the thinness of digital interactions does not seem sufficient to support the kind of rich interaction within the same shared space that characterizes traditional communities. However, prior work has shown that there are strong social commitments and ties between participants, particularly once they become more involved and begin attending in‐person events and meetups (Coleman & Hill, 2005; Kelty, 2008; Sarma & Matheus, 2015). Such work has studied FOSS—and other “commons‐based peer‐production projects” (Benkler, 2008) like Wikipedia or community science^1^—as a “community of practice” (COP; Lave & Wenger, 1991; Wenger, 1999).

In CoPs, members' social bonds are established through learning to participate in compatible ways. In this sense, the success of FOSS and related peer‐production projects begin with the thinness of being able to contribute to a project by posting a short message about a bug or by uploading a few lines of code modifications to make a “pull request.” Such work often uses Lave and Wenger's (1991) related concept of “legitimate peripheral participation” within a CoP. This work has focused on how successful membership and socialization in such projects begin with more lightweight and low‐risk activities; if newcomers successfully participate, they are given more opportunities for more complex activities for more complex activities, as has been found in other peer‐production communities like community science (Mugar, Østerlund, Hassman, Crowston, & Jackson, 2014), fan fiction (Fiesler, Morrison, Shapiro, & Bruckman, 2017), and other FOSS projects (Gasson & Purcell, 2018).

#### 1.1.2 Characteristics and culture of FOSS contributors

FOSS contributions are largely considered “donations of labor” (Geiger et al., 2021, p. 10). Recent studies and surveys suggest that around half of FOSS contributors receive some kind of financial compensation for their activity on FOSS projects (Nagle, Wheeler, Lifshitz‐Assaf, Ham, & Hoffman, 2020; Riehle, Riemer, Kolassa, & Schmidt, 2014), although differences in payment by *maintenance* and *development* exist (Geiger et al., 2021). The vast majority of paid contributors are compensated as part of activities for their primary employers, rather than through mechanisms designed to purely support FOSS development (e.g., crowdfunding, grants). However, even paid contributors also donate their labor to projects above and beyond their employer‐related contributions (Riehle et al., 2014).

As companies and other organizations rely increasingly on FOSS, these organizations sometimes encourage or even explicitly compensate their employees to work on FOSS development (Germonprez, Link, Lumbard, & Goggins, 2018; O'Neil, Muselli, Raissi, & Zacchiroli, 2021). On the other hand, not all companies that rely on FOSS projects give back to those projects. This often leads to difficulties for maintainers who struggle to cope with the additional demands of the company and its users in their community (Geiger et al., 2021).

Unsurprisingly, the question (and sources) of funding can introduce new complexities and difficulties in the maintenance and scale of FOSS projects (Geiger et al., 2021). Some early work identified differences in interaction patterns between paid and volunteer contributors (Herraiz, Robles, Amor, Romera, & González Barahona, 2006), but recent work finds that the distinction has become much less salient within the community (Germonprez et al., 2018). Current research does not provide insight into the reasons why this shift may have happened. It may be due to the normalization of paid labor in FOSS, to the increased size and distribution of FOSS communities, to the mainstreaming of FOSS generally, or to the lack of signaling of status (paid or volunteer)—or none, some, or all of these and more.

#### 1.1.3 Visibility and salience of social dynamics on GitHub

One of the earliest ethnographic studies of GitHub focused on the interconnectedness between the work and socioemotional components of public FOSS development (Dabbish et al., 2012). FOSS contributors recognized GitHub's essential role in helping contributors work together effectively on code, but the open nature of the platform created “social translucence” (Erickson & Kellogg, 2000), supporting the awareness of developers' behavior and making them more accountable for it. Developers reported feeling that it simultaneously opened them up to negative social repercussions (e.g., being judged for programming ability) and pressured them to engage in positive social interactions with others (e.g., thanking them).

This early work also highlighted the incredible amount of information that users infer from other users' patterns and content of communication and contribution (Dabbish et al., 2012). A specific project's responsiveness to external contributions of code was a particularly important and highly visible marker for people outside of the project, especially those looking for new projects to which they could contribute. Given the acute awareness of others' behavior and the accompanying implicit (or explicit) expectations for social behavior, specific FOSS projects may develop their own (healthy or unhealthy) norms for acceptable social behavior within their developer communities. This would lead to the enculturation of new users into the community based on their early exposure to that community's social behavior, which—in turn—shapes those new community members' own behavior within the community (Port, 2010).

#### 1.1.4 Positive emotions lead to positive outcomes

Software developers' emotional lives and dynamics are critical to their work. They are highly aware of their teams' social dynamics, including divisions of responsibilities (de Magalhães, 2017). Motivation to continue is related to pride and recognition, while satisfaction with the work—a driving factor in continued engagement—has been closely tied to happiness and positive emotional states (França, Sharp, & Da Silva, 2014). General positive emotions have been tied to better analytical problem‐solving (Graziotin, Wang, & Abrahamsson, 2014) and self‐assessed productivity (Graziotin, Wang, & Abrahamsson, 2013). Work‐related positivity is connected to better work‐related outcomes (Shaw, 2004). Unhappiness, by contrast, leads to withdrawal from the work (Graziotin, Fagerholm, Wang, & Abrahamsson, 2017).

#### 1.1.5 Contributing to learn, learning to contribute

Learning to participate in software development is a common motivation for FOSS contributors (Storey, Zagalsky, Figueira Filho, Singer, & German, 2016). This often too has a latent social component: Many are specifically interested in learning through feedback from experienced programmers (Dabbish et al., 2012). Social recognition and community impact are also common motivations (Santos et al., 2017). These findings mirror other findings on volunteering generally (Clary, Snyder, & Ridge, 1992) and on software engineers' motivations specifically (Santos et al., 2017). Given the drive to learn through community engagement, social norms are likely to encourage newcomers to start by working with reporting bugs until they gain the experience needed to contribute code (Storey et al., 2016), implying an enculturation process guiding interaction dynamics (Port, 2010; Rączaszek‐Leonardi, 2010).

#### 1.1.6 Social support is critical for FOSS newcomers

As we have already discussed, the community's social fabric is important for software engineers and FOSS contributors more generally, but social‐emotional dynamics are especially important for FOSS newcomers. Newcomers to FOSS projects face a number of technical and logistical challenges (Steinmacher, Silva, & Gerosa, 2014; Von Krogh, Spaeth, & Lakhani, 2003), but the social relations between newcomers and existing members of a project pose special concerns for those making their first FOSS contributions (Steinmacher, Conte, Gerosa, & Redmiles, 2015; Steinmacher, Wiese, Conte, Gerosa, & Redmiles, 2014). For example, newcomers may not feel that their contributions are well‐received or well‐supported by established members of the community, especially if they are not thanked or if they do not receive timely responses (Steinmacher et al., 2015; Steinmacher, Wiese et al., 2014). However, a newcomer's perceived rudeness can also be a barrier to integration into the community (Steinmacher et al., 2015).

### 1.2 An overview of GitHub

The present work analyzes community data from GitHub (http://www.github.com), an online platform designed to facilitate code exchange, code sharing, and collaborative code development. Each software project on GitHub has its own *repository* (or repo), which contains the full history of all files in a directory structure, with all changes tracked and indexed. While authorized members of a repository are (by default) able to immediately *merge* (or update the repository's official files) a *commit* (or a proposed change to something in the repository) to include their code changes, all other GitHub users can make a *pull request* (or PR), asking for their proposed code changes to be merged into the repository. Every new PR creates an open discussion thread.

Many projects—especially larger ones—require all PRs to be reviewed before being merged. This usually involves getting approval from one or more project members who review the PR according to various project‐specific rules and norms. Pull requests can be quick decisions with little to no discussion or can be drawn out over dozens or even hundreds of comments over months, which generally unfold in the associated PR discussion thread.

GitHub also lets any user file an *issue* in the repository's issue tracker. Issues are used for discussing bug reports, feature requests, hypothetical PRs, and tech support. Practically, PRs and issues can be strongly linked: Often, a PR will refer to a specific issue that the proposed change resolves. PRs, issues, and commits are uniquely numbered, and the discussion platform makes it easy to link to any of these in an issue or PR discussion thread.

Issues and PRs share similar structures. Each is an ordered set of comments, headed by the text of the issue or PR. The web interface orders comments reverse chronologically and displays the commenter's username, profile image, time of the comment, and whether they are a project member or (starting in July 2017) a first‐time contributor to the repo.

We chose GitHub because its integrated communication and code development gives us a uniquely expansive view of the lifecycle of open‐source software. While not every single act of communication in a FOSS project hosted on GitHub takes place exclusively through GitHub, many projects have strong norms or policies encouraging as much communication to take place on GitHub as possible. Although projects often specify unique expectations about communication for their members and newcomers, the issue tracker and PRs are the default spaces for members and non‐members to raise issues and discuss changes.

### 1.3 Language and interaction from the dynamical approach

Many areas of cognitive science have grown to incorporate elements of *dynamical systems theory* (DST) over the past 50 years or so. While dynamical systems theorists across fields have different definitions of dynamical systems, the dynamical approach in cognitive science can generally be described as a commitment to the idea that perception, (inter‐)action, and cognition can best be described as systems that change over time (van Gelder, 1998). The dynamical approach in cognitive science has been extensively discussed elsewhere (e.g., Beer, 2000; Chemero, 2011; van Gelder, 1998); here, we specifically take the approach of language and social interaction as dynamical systems.

Dynamical systems (or *complex adaptive systems*) approaches to language and interaction have spanned temporal and participatory scales, from the production of a single utterance to the evolution of human language capabilities (e.g., Five Graces Group et al., 2009; Rączaszek‐Leonardi & Kelso, 2008; Richardson, Dale, & Marsh, 2014; Vallacher, Read, & Nowak, 2002). As with the DST approach more generally, views differ about what a dynamical approach to social communication should mean. Here, we intend it to mean that language and interaction patterns emerge (or develop within the system without a priori planning) from a variety of individuals (with different views, personalities, and abilities) interacting with one another in specific local contexts (including specific physical or virtual locations, specific intentions or goals in mind, and specific social or community pressures). The individual (inter‐)actions of and between community members can be seen as the bottom level of the system, operating at the fastest timescale; the community's emergent social and linguistic phenomena can be seen as the highest level of the system, operating at the slowest timescale. These two scales—the individual and the community—are deeply and inextricably linked: The specific interactions both shape and are shaped by the community‐level phenomena, and the community‐level phenomena constrain and are sustained by the specific individual interactions. This spans timescales from minutes to years and participatory scales from two to thousands of people. (See Paxton, Dale, & Richardson, 2016, for more on this conceptualization.)

Such a dynamical approach entails that language is necessarily communal, pragmatic, and situated. Language belongs to *communities*, not to individuals (Port, 2010). As such, language use specifies the individual within their community, communicative context, and social goals (Hodges & Fowler, 2010). Specific language structures emerge from interacting communities at varying timescales. For example, referential conventions appear within the span of an interactive psycholinguistics experiment (Hawkins, Goodman, & Goldstone, 2019), perceptual labels appear within the span of enculturation in online communities (Danescu‐Niculescu‐Mizil, West, Jurafsky, Leskovec, & Potts, 2013), and dialect‐specific pronunciations appear over generations (Port, 2010). The speed and efficacy of interpersonal communication shape the evolution of community‐specific language structures from grammatical structures to concepts (Rączaszek‐Leonardi, 2010). However, these multiple timescale interactions are notoriously difficult to investigate in controlled laboratory experiments, leading to a push for investigations of naturalistic language use through real‐world data (Rączaszek‐Leonardi & Kelso, 2008).

### 1.4 The present study

The current work tests theories of dynamical social interaction in a specific, real‐world application. We aim to better understand the dynamics of communication by focusing on the time‐ and context‐changing language of communities with well‐documented activity. To do this, we study the naturally occurring communication patterns of FOSS contributors as they develop and maintain codes on GitHub. Our findings may provide insights to improve these communities' sustainability and empirically support a dynamical systems view of language.

#### 1.4.1 Applied goals

Certain properties of language may be particularly important indicators of community health—specifically, *sentiment* (or emotion)^2^ and gratitude, given the importance of these to individual and interpersonal processes to social health generally and within software programming communities (Algoe, 2012; Dabbish, Stuart, Tsay, & Herbsleb, 2012; Emmons & Shelton, 2002; França, Sharp, & Da Silva, 2014; Steinmacher, Conte, Gerosa, & Redmiles, 2015; Yoshimura & Berzins, 2017). However, consistent with a dynamical systems approach to language (Gibbs & Van Orden, 2012; Hodges & Fowler, 2010; Paxton, Dale, & Richardson, 2016), these patterns should be sensitive to the specific *context* in which communication occurs (Mäntylä, Adams, Destefanis, Graziotin, & Ortu, 2016; Rączaszek‐Leonardi & Kelso, 2008) and the person's *own relationship* with the community (and, by extension, their enculturation into the community; Thorne, Black, & Sykes, 2009; Rączaszek‐Leonardi, 2010). Therefore, patterns should vary by the community (Port, 2010; Thorne et al., 2009) and over time (i.e., as members join and leave; Demjén, 2018; Rączaszek‐Leonardi & Kelso, 2008).

We analyze naturally occurring data or “trace data” (i.e., data left behind as people interact with the physical or digital world) in eight FOSS communities as they developed pivotal software *projects* on GitHub. We selected this “purposeful sample” (Palinkas et al., 2015) of projects based on the following criteria: All develop widely relied‐upon digital infrastructure, primarily use GitHub issues and pull requests to collaborate, have existed for at least 3 years, have over 50 contributors with more than five contributions each, and have over 100 contributors total. However, projects have a wide variation in terms of their longevity (founded in 2001 to 2014) and community size (number of members from 90 to 6,806). For consistency, we only selected projects using the same programming language (Python) but included projects supporting a number of different kinds of activities, from visualization and software documentation to basic math and machine learning. This range allows us to examine the general dynamics of online software developer communities and the impact of those dynamics on community health.

Specifically, we examine the ways in which communication context shapes language use. For the present work, we define *communication context* as the type of GitHub activity in which the user engaged: posting an issue (e.g., flagging a bug, requesting a new feature), posting a PR (i.e., proposing new content or a fix to the code), commenting on an issue (i.e., adding to the discussion of a posted issue), or commenting on a PR (i.e., adding to the discussion of a posted PR). From the contributor's free‐form language, we quantify two properties of language. The *sentiment* is measured with natural language processing techniques that score positive, negative, and neutral language, which we averaged into a single sentiment score from (−1 [entirely negative] to +1 [entirely positive]) for each activity. We measured *gratitude* for each activity as the log of the simple count of gratitude words in the text. *Community membership* of the individual providing each activity (i.e., whether a given individual was a member or non‐member of the project) was assessed dynamically: At the time of each activity, a person was only labeled a “member” if they had contributed five or more posts to that community.^3^

After identifying these patterns of communication, we next examine whether a community's ability to retain new members is linked to the community's language dynamics and responsiveness in response to newcomers. For example, within Wikipedia, first‐time volunteer contributors were more likely to contribute again if they received friendlier and more personalized welcome messages (Geiger, Halfaker, Pinchuk, & Walling, 2012). However, given the variety of goals that software engineers have when contributing to FOSS projects, we again expect to see signs of context sensitivity: Newcomers who give different types of contributions should be motivated to return based on different kinds of community responses.

#### 1.4.2 Theoretical goals

In addition to providing specific applied insights into the community dynamics underpinning critical digital infrastructure, the present work also targets specific theoretical goals in the dynamical systems approach to language and social interaction. Building on previous calls (Rączaszek‐Leonardi & Kelso, 2008), we here use naturally occurring data to investigate language as a dynamical system in real‐world communities. We investigate the differences in language structures across communities, the changes in those language structures over time, and the impact of these behaviors on the community as a whole. In other words, from the DST framing, we are able to study multiple scales of the system simultaneously and within a real community—the language use patterns of individuals conducting specific activities, the distinctiveness of those patterns within specific communities, and the effectiveness of those patterns in promoting the health of the community itself.

We also extend previous investigations of dynamical language analyses to include a wider range of language structures. More canonical language structures for the dynamical approach include structural components (e.g., grammar; Port, 2010) or more traditionally cognitive components (e.g., concepts; Rączaszek‐Leonardi & Kelso, 2008). Here, we extend this to include more explicitly social language structures by targeting sentiment and gratitude, which we see as complementary to community‐level studies of lexical‐conceptual changes (e.g., Danescu‐Niculescu‐Mizil et al., 2013).

## 2 Method

All codes for data preparation and analysis are available at the GitHub repository for our project: https://github.com/a‐paxton/oss‐community‐health. All data for the analyses are available on OSF: https://osf.io/6ncwt/.

### 2.1 Corpus and data preparation

For the present study, we analyzed posts (i.e., original issues and original PRs) and comments (i.e., comments on issues and comments on pull requests)—together denoted as “activities” moving forward—in eight projects on GitHub: matplotlib, mayavi, numpy, pandas, scikit‐image, scikit‐learn, scipy, and sphinx‐gallery. All projects rely on the Python programming language and emerged from the scientific Python community. These projects provide support for a number of vital scientific activities—from documentation to machine learning—and are accordingly essential both to specific scientific projects and to other Python packages. For summary statistics on these projects, see Supplementary Table 7 in the Supporting Information.

Data were collected from GitHub's API using watchtower, a Python library. We assembled a dataset of the following for each activity: unique activity identifier, author name, author's membership status in the community at the time of activity, activity type (i.e., original issue, original PR, comment on issue, comment on PR), associated original activity identifier (for comments), text body, date created, date edited (if applicable), and date resolved. We downloaded all records created and updated before January 1, 2019. The dataset comprises 19,430 unique users; 36,538 posted pull requests; 253,495 comments on PRs; 32,133 posted issues; and 185,529 comments on issues.

Because the data are naturally occurring corpus data, we needed to extensively clean them before moving forward with analyses. We filtered out all activities associated with bots to ensure that our analyses only targeted actual community members. For each activity, we first removed any text not viewed by the user; projects often use templates that include hidden HTML elements in the body of the activity that is often automatically generated by the template. We then annotated each activity with positive, negative, and global sentiment scores using vaderSentiment (Hutto & Gilbert, 2014), a sentiment analysis tool that is designed to capture affective features of language in online contexts. Importantly, it captures both standard affect words and novel affect features of computer‐mediated conversation (e.g., UTF‐8 encoded emojis, punctuation, capitalization). Each activity was also annotated with the number of words associated with gratitude, compiled with traditional words of gratitude and online expressions of gratitude (i.e., *appreciate*, *appreciated*, *appreciative*, *grateful*, *gratitude*, *indebted*, *obliged*, *thank*, *thanks*, *thankful*, *thanx*, *thx*, *tks*).

**Fig. 1:**
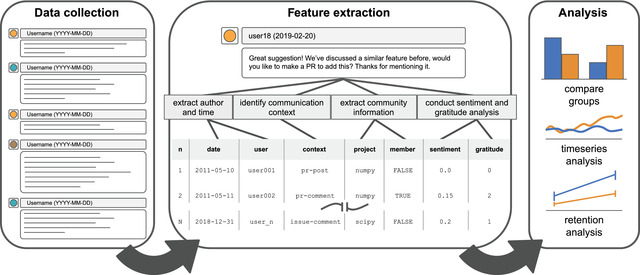
Diagram of data collection, feature extraction, and analysis workflow for the current project. Records of activity were downloaded from GitHub for eight free and open‐source software projects. We extracted a series of features from each activity record, including basic metadata (i.e., the date it was created, the user who created it), communication context (i.e., whether someone was posting a PR, commenting on a PR, posting an issue, or commenting on an issue), community metrics (i.e., the project to which it belonged, whether the user was a member of the community or not), and language analysis (i.e., extracting a sentiment score and a count of gratitude words). Data were then subjected to a series of analyses, including comparing the overall behavior of groups, tracking of communities' language use over time, and quantifying factors related to newcomer retention.

**Fig. 2:**
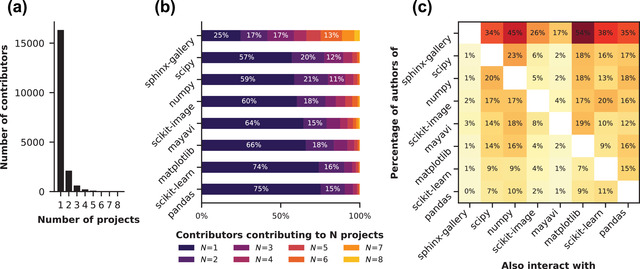
Panel A shows the distribution of the number of projects an author contributes to. Panel B shows the percentage of authors per project that contributes to 1,2,…, *N* projects studied in this paper. Panel C shows the percentage of authors for each project that contributes to one of the other projects studied in this paper.

Each activity made by each individual was annotated to indicate whether their author is a “member” of the repository's community at the time of the posting. Rather than relying on GitHub's membership (which is not exclusively determined at the repository level), we identified a member being someone who has contributed at least five posts.^4^ We also identified and specially annotated the first post in each community made by each author. (See Fig. 1 for visualization of analysis and Fig. 2 for visualization of the community members.)

### 2.2 Computational analysis

#### 2.2.1 Effect of community membership on sentiment

We evaluated whether sentiment differed between members and non‐members of a community. We modeled sentiment of each activity, $s_{j}$:

(1) $$s_{j}=\mu_{m_{j}}+\mu_{a_{j}}+\mu_{p_{j}}+\varepsilon_{j},$$

where $\mu_{m_{j}}$ is the average sentiment of an activity of a particular group. Sentiment of individual activities deviates from this average by a random effect capturing author sentiment score $\mu_{a_{j}}$ and a random effect capturing project sentiment score $\mu_{p_{j}}$. Remaining sources of random errors are modeled by $\varepsilon_{j}$. Statistical significance is computed through a *t*‐test on $\mu_{m_{j}}$.

#### 2.2.2 Effect of activity type on sentiment

We assessed whether activity sentiment differed according to type: “Opening an issue” (post:issue), “Opening a pull request” (post:PR), “Commenting on an issue” (comment:issue), and “Commenting on a pull request” (comment:PR).

(2) $$s_{j}=\mu_{t_{j}}+\mu_{a_{j}}+\mu_{p_{j}}+\varepsilon_{j},$$

where $\mu_{t_{j}}$ is the average sentiment of an activity for a particular type of activity. We then assessed whether the sentiment of each activity type differs by performing *t*‐tests with the following null hypotheses:

- Does sentiment vary between posts and comments on issues? $H_{0}:\mu_{\text{post:issues}}=\mu_{\text{comment:issues}}$
- Does sentiment vary between posts and comments of PRs? $H_{0}:\mu_{\text{post:PR}}=\mu_{\text{comment:PR}}$
- Does sentiment vary between posts of issues and PRs? $H_{0}:\mu_{\text{post:issues}}=\mu_{\text{post:PR}}$
- Does sentiment vary between comments on issues and PRs? $H_{0}:\mu_{\text{comment:issues}}=\mu_{\text{comment:PR}}$

#### 2.2.3 Effect of activity type and community membership on sentiment

We then assessed whether the interaction between activity type and community membership had an effect on an activity's sentiment:

(3) $$s_{j}=\mu_{\left(t_{j},m_{j}\right)}+\mu_{a_{j}}+\mu_{p_{j}}+\varepsilon_{j},$$

where $\mu_{\left(t_{j},m_{j}\right)}$ is the average sentiment of an activity of a member group $m_{j}$ (member, non‐member) and an activity type $t_{j}$ (comment:PR, comment:issue, post:PR, post:issue). We can now assess whether the tests performed above hold regardless of community membership.

#### 2.2.4 Project variation in the effects of activity type and community membership on sentiment

We test whether the results observed above vary across different projects:

(4) $$s_{j}=\mu_{\left(t_{j},m_{j},p_{j}\right)}+\mu_{a_{j}}+\varepsilon_{j},$$

where $\mu_{\left(t_{j},m_{j},p_{j}\right)}$ is the average sentiment of an activity of a particular author group, activity type for project $p_{j}$. Sentiment from individual activities deviates from this average by a random effect sentiment score $\mu_{a_{j}}$, and the remaining sources of errors are modeled by $\varepsilon_{j}$. This assesses whether the sentiment differs not only by community membership and activity type but *also* across individual projects by performing the hypotheses tests described above for each project independently.

#### 2.2.5 Correcting for multiple tests

We corrected for multiple tests with Benjamini–Hochberg.

#### 2.2.6 Studying gratitude

We repeated the analyses described above on measures of gratitude.

#### 2.2.7 Exploring sentiment and gratitude over time

We next quantified interproject variability of sentiment and gratitude over time. We separated activities by year created. To identify projects variability in sentiment over time, we estimated for each activity type t, year y, and membership group m whether the average sentiment for project p was significantly different than the average sentiment of all other projects. We thus modeled the sentiment of each activity, $s_{j}$:

(5) $$s_{j}=\mu_{\left(t_{j},m_{j},y_{j},{\hat{p}}_{j}\right)}+\mu_{a_{j}}+\varepsilon_{j},$$

where ${\hat{p}}_{j}$ indicates whether activity j belongs to the project of interest p or not. We then assessed for each project whether the average sentiment of an activity type and membership type in that year differed from the rest of the projects by performing *t*‐tests. We corrected for multiple tests using Benjamini–Hochberg. The process was repeated for parallel analyses of gratitude.

#### 2.2.8 Effects of language dynamics on newcomer retention

Finally, we explored the effect of language dynamics and other variables on newcomer retention. For each project, we extracted every author's first contribution to the project, counting only posts (i.e., issue posts or PR posts). We then labeled each author i as whether they were “retained” or not ($r_{i}\in \left\{0,1\right\}$)—in other words, whether they returned to make a second contribution. Importantly, newcomers were determined on a project‐by‐project basis, not on a global scale (i.e., a person could have already been an active member of community A when they are counted as a newcomer for community B).

We thus studied the effect of the following variables on the likelihood that a person would return to contribute: $c_{i}$, post type (issue or PR); $d_{i}$, the amount of time (in days) that the post remained open; $g_{i}$, the cumulative gratitude unigram count used in the comments to the post; $s_{i}$, the mean sentiment of the comments to the post; $h_{i}$, the sentiment score of the most positive comment to the post; $n_{i}$, the sentiment score of the most negative comment to the post; $x_{i}$, the total number of comments made on the post; and $y_{i}$, the ratio of members to non‐members among replies to the post.

For assessing the effect of contribution type, the only categorical variable of interest, we modeled the likelihood $r_{i}$ that a person would return to contribute as follows:

(6) $$\mathrm{logit}\left(Pr\left(r_{i}\right)\right)=\mu_{c_{i}}+\mu_{p_{i}}+\varepsilon_{i},$$

where $\mu_{c_{i}}$ is the average sentiment of a contribution type and $\mu_{p_{i}}$ is a random effect capturing the likelihood to return for a specific project. We then performed a *t*‐test to assess whether an author was more likely to return if their first contribution was a PR or a ticket.

For all of the other variables of interest, which are continuous, we modeled the likelihood for an author to return as follows:

(7) $$\mathrm{logit}\left(Pr\left(r_{i}\right)\right)=\beta_{0}^{v}+\beta_{1}^{v}v_{i}+\mu_{p_{i}}+\varepsilon_{i},$$

where v is the variable of interest (amount of time d, cumulative count of gratitude language used in the comments g, mean sentiment of comments to the post m, ...). $\beta_{0}^{v}$ thus captures the intercept of the linear model, while $\beta_{1}^{v}$ the slope, that is, the effect of the variable v on the model. We then performed a *t*‐test on the estimate $\hat{\beta_{1}^{v}}$.

We further explored interaction terms between the categorical variables $c_{i}$ and the continuous variables listed above by modeling the likelihood of an author returning as

(8) $$\mathrm{logit}\left(Pr\left(r_{i}\right)\right)=c_{i}\cdot \left(\beta_{0}^{v}+\beta_{1}^{v}v_{i}\right)+\mu_{p_{i}}+\varepsilon_{i},$$

thus resulting in separate linear models estimated for PRs and issues. We performed a *t*‐test on the estimates of the slope to assess whether a variable had a significant effect. We added another predictor—the variance of the comment sentiment—and its interaction with contribution type in a follow‐up analysis that we present in the Results section. We corrected for multiple hypothesis testing with Benjamini–Hochberg.

## 3 Results and discussion

For clarity and flow, all statistics are provided in tables. Additional figures showing individual results are available in our online Supporting Information: https://osf.io/6ncwt/.

### 3.1 Language is constrained by context and identity

**Fig. 3:**
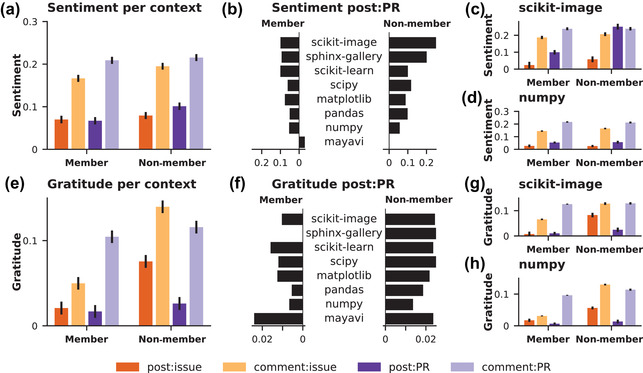
Summary visualizations of message sentiment (panels A and B) and gratitude (panels E and F) by context and membership. Community‐specific visualizations are provided for scikit‐image (panels C and D) and numpy (panels G and H). Context indicated by color: posted issue (dark orange), comment on an issue (light orange), posted PR (dark purple), or comment on a PR (light purple). Membership indicated by position: member (left panels) or non‐member right panels).

Our first set of analyses explored the ways in which community membership and communication context shapes language patterns (see Fig. 3 and Supplementary Tables 1 and 2 in the Supporting Information).

#### 3.1.1 Sentiment differs by activity and community membership

Overall sentiment differed significantly by activity. Generally, comments were more positive than posts, and PRs were more positive than issues. Without considering activity type, community members and non‐members did not systematically use sentiment differently. However, systematic differences in sentiment appeared in the ways that members versus non‐members use language in *certain kinds* of activities. Overall, members and non‐members do not significantly differ in their sentiment for issues‐related activity nor in their comments on pull requests, but we do see that non‐members are significantly more positive than members when they post PRs. Interestingly, we see that the overall effects of activity type hold across members and non‐members except in the sentiment of non‐members' comments; we find no significant difference between non‐members' comments on issues and non‐members' comments on pull requests.

#### 3.1.2 Expressions of gratitude vary strongly by activity and community membership

Unlike overall sentiment, membership status predicts the amount of gratitude expressed in posts: Non‐members express gratitude significantly more than members. While the amount of gratitude expressed does not differ between posted issues and comments on issues, gratitude is expressed more in comments on PRs than in posted PRs, more in posted issues than in posted PRs, and more in comments on PRs than in comments on issues.

We found significant differences in interaction effects, suggesting that both community membership and activity type presented distinct constraints on gratitude expression. We found only three non‐significant comparisons: Non‐members and members were not significantly different in the gratitude expressed in posted PRs; members did not show significant differences in gratitude when posting an issue or posting a PR; and non‐members did not use significantly different gratitude language when commenting on an issue or a PR.

#### 3.1.3 Communities differ in sentiment and expressions of gratitude

Although some communities exhibit dynamics identical to the aggregate analyses, we do see systematic differences across specific communities. For example, pandas members use more positive language when posting issues than PRs and show higher positivity when commenting on PRs than non‐members. Interestingly, some communities show lower (i.e., numpy, scikit‐image, scikit‐learn) or no (i.e., matplotlib, mayavi, sphinx‐gallery) systematic differences between members and non‐members. However, the majority of communities have more or fewer interaction effects than the aggregate—showing, in other words, that some communities' language dynamics vary widely by membership while other communities' language dynamics are equivalent across membership status. These results give us the first glimpse that the differences in language are not due simply to a user's status within the community but are, in fact, differences that emerge based on the ways that community membership constrains language use in specific contexts.

As with sentiment, gratitude dynamics showed project‐specific effects that support our hypothesis that community‐specific dynamics constrain the expression of gratitude. The general model demonstrated a fair amount of specification by membership and activity type, but only one community mirrored the specification observed at the aggregate level (i.e., scikit‐learn), with others being more specified (i.e., scikit‐image, matplotlib, pandas, numpy, scipy) or less specified (i.e., mayavi, sphinx‐gallery). Interestingly, two communities with less specification differed greatly in their amounts of gratitude language—with mayavi being very high and sphinx‐gallery being very low. However, not all communities' gratitude dynamics moved in the same direction across activities. For example, contrary to the general pattern, matplotlib's members use more gratitude language when posting issues than when posting PRs; mayavi's members use more gratitude language than non‐members when commenting on PRs; and numpy's non‐members used more gratitude language when commenting on an issue than a PR.

Overall, the differences in results between the sentiment and gratitude models highlight the distinct language dynamics that each measure captures—with, for example, some of the lowest‐sentiment communities demonstrating very high gratitude.

### 3.2 Different aspects of language unfold differently over time

**Fig. 4:**
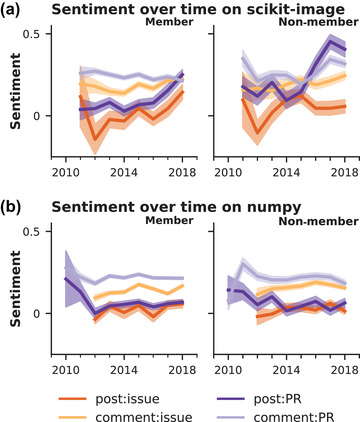
Model fits for analysis of sentiment over time by context and membership for scikit‐image (panel A) and numpy (panel B). Context indicated by color: posted issue (dark orange), comment on an issue (light orange), posted PR (dark purple), or comment on a PR (light purple). Membership indicated by position: member (left panels) or non‐member right panels).

Like any dynamical system, community dynamics should evolve over time (Demjén, 2018). Our next set of models explored the degree to which specific projects showed unique context‐ and membership‐specific dynamics over time (see Fig. 4 and Supplementary Tables 3 and 4 in the Supporting Information).

#### 3.2.1 Sentiment shows patterns of coherence and distinctiveness over time

Communities showed distinctive patterns of language dynamics over time. One community (scikit‐image) showed little deviation from the aggregate, while another (numpy) showed consistently more negative language by members across activity types over time. However, most communities show only some deviation from the aggregate. For example, most communities with significantly more negativity tend to do so only in one of the four activity types (i.e., recent increase in negativity for member issue comments for sphinx‐gallery and mayavi, consistent negativity for member PR comments in matplotlib). Three communities historically showed more positivity among members overall (i.e., scipy, scikit‐learn) or in specific activities (i.e., pandas), although one of these recently became less distinctive in its positivity (i.e., scikit‐learn).

Non‐members also showed distinct community‐specific dynamics, although to a lesser extent. Of those that differed significantly from the aggregate, we generally see non‐members being more positive. Some show consistently more positive sentiment in all activities (i.e., scipy) or PR‐related activity (i.e., scikit‐image). Others show changing trends in recent years: pandas shifted from being more positive in comments on issue posts, while scikit‐learn interestingly transitioned from displaying aggregate behavior to deviating from the aggregate, becoming more positive in all comments but more negative in all posts. Only two communities showed significantly more negative dynamics than aggregate, with matplotlib demonstrating recent spikes in negativity in specific activity types (i.e., PR comments) and numpy—similar to the members of this community—showing significantly and consistently more negative sentiment.

#### 3.2.2 Gratitude varies less by time than by community

Four communities showed consistent deviations in gratitude language from the group across the observation period: numpy members consistently used lower rates of gratitude language in issue comments; scipy members consistently used more gratitude language in PR comments; pandas members consistently used more gratitude language in issue posts; and, interestingly, matplotlib members consistently used more gratitude in their issue comments and PR posts but less gratitude in their PR comments. Two communities changed over time: mayavi members became more grateful in PR comments, while scikit‐learn members fell from higher to lower relative rates of gratitude language overall.

For non‐members, fewer communities showed different gratitude dynamics from the general trend. This is relatively unsurprising given that non‐members should be shaped less by the community‐specific dynamics. However, we do see some shifting trends over time: Four communities showed a relative increase in gratitude from the aggregate for issue activity (i.e., sphinx‐gallery, mayavi) or comments during the last several years (i.e., numpy, scipy); one community shifted from a history of higher relative gratitude expression in issue comments to lower relative gratitude expression in issue posts (i.e., pandas); and one community began to have lower relative gratitude in nonmember *and* member comments (i.e., scikit‐learn).

### 3.3 Contribution‐specific responses to newcomers shape the likelihood of returning

**Fig. 5:**
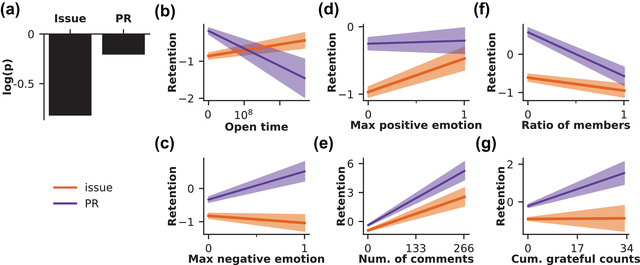
Model fits of predictors of newcomer retention. Panel A shows the likelihood of a newcomer returning based on the type of their first contribution. The remaining panels show the model fits of the continuous predictors by contribution type (purple = PR; orange = issue): the amount of time in days between the opening of the contribution and its resolution by the community (panel B), the negative sentiment score of the most negative comment in response to the contribution (panel C), the positive sentiment score of the most positive comment in response to the contribution (panel D), number of comments posted in response to the contribution (panel E), the ratio of members to non‐members who posted comments in response to the contribution (panel F), and the cumulative count of gratitude words among comments posted in response to the contribution (panel G). For panels B–G, the trend lines reflect the extent to which each variable (shown on the *x*‐axis) impacts retention (shown on the *y*‐axis).

In our final set of analyses, we investigated the impact of language dynamics and community responsiveness on the likelihood that a first‐time contributor will come back to post a second time (see Fig. 5 and Supplementary Table 5 in the Supporting Information). The modal outcome, of course, is that newcomers do *not* come back: Only 35.01% of first‐time contributors return to make a second contribution. However, this analysis seeks to understand how the community's response to newcomers could make them more or less likely to return. Although these communities have other interactions outside of GitHub, their responses to newcomers will often be the only point of contact—and, therefore, potential recruitment or distancing—that these newcomers have with the community.

Generally, newcomers whose first post was an issue were significantly less likely to return, while newcomers whose first post was a PR were significantly more likely to return. Importantly, the majority of newcomers (70.50%) in our dataset contributed an issue. The structure and content of comments on newcomers' posts were also influential: More positive, more grateful, more commented upon, and less member‐heavy comment threads were more predictive of newcomers returning. Neither open time nor maximum negative sentiment significantly predicted retention.

As with previous models, these effects were modulated by interactions with post type. Two factors—more comments and less member‐heavy comment threads—were both predictive for issues and PRs, but the remaining effects differed between post types. Newcomers who approached the community with a potential problem to be solved (i.e., issues) were more likely to return when greeted with higher average positivity. Newcomers who approached the community with a potential contribution to the code (i.e., PRs) were more likely to return when they had more maximally negative comments and more gratitude throughout the comment thread. Interestingly, the effect of time until post closure diverged, with a longer delay predicting newcomer retention when posting issues and a shorter delay predicting newcomer retention when posting PRs. In other words, *communication context*—especially, in our case, the *motivation* driving a newcomer's contribution—is vitally important to shaping the emergence and impact of language.

#### 3.3.1 Hints of differing motivations across newcomers

The opportunity for learning from more experienced programmers has been noted as one of the motivations driving FOSS contribution in previous qualitative work (Dabbish et al., 2012; Storey et al., 2016), and the seemingly contradictory pattern of results for sentiment and gratitude across PRs and issues may provide evidence to support that. Specifically, it may be that newcomers who are contributing new code are motivated to return when they receive constructive criticism *and* appreciation from the community and that newcomers who are flagging potential problems in the code are simply looking for recognition and validation in their bug‐finding activities (cf. Storey et al., 2016).

We tested this hypothesis by examining two additional predictors of retention—sentiment of the most positive comment in the thread, sentiment variance in the comment thread—and their interactions with post type (see Supplementary Table 6 in the Supporting Information). In these exploratory analyses, we expected to find that sentiment variance would be associated with higher retention for newcomers submitting PR (but not issues) and that maximum positive sentiment would be associated with higher retention for newcomers submitting issues (but not PRs). Contrary to our expectations, we found no significant effect of variance or its interaction with post type. However, consistent with our expectations, we did find a significant interaction effect of maximum positive sentiment (along with a significant overall effect of maximum positive sentiment): Newcomers who posted issues (but not PRs) and were met with a more positive response from the community were more likely to return.

### 3.4 Implications for FOSS communities

Our findings have direct implications for FOSS communities. First, our findings of community‐specific differences may inform contributors and community leaders as consider recruitment and retention. Each community has different communication dynamics, and even if newcomers to a given project have contributed to another FOSS project, it may take time to learn (implicitly or explicitly) the norms of a new community.

Second, newcomers who were engaged in generating a new code were more likely to return, contrary to perceptions about the process of newcomer onboarding (Storey et al., 2016). While this effect may be driven by other factors (e.g., programming skills), communities may leverage this finding to their benefit by highlighting simple ways that newcomers can contribute.

Third, community responsiveness was important to newcomer retention, but *ideal* responsiveness depended on the type of newcomer engagement. FOSS communities may better retain newcomers by aligning feedback to the contribution: by providing constructive but supportive feedback about code to those trying to contribute to the codebase and by giving validation and encouragement to those trying to point out potential problems with the existing codebase.

At an individual level, FOSS contributors may consider changing their interaction dynamics with other people to promote newcomer retention to improve general supportiveness, appreciation, and constructive criticism. Dynamical systems are a product of top‐down and bottom‐up forces, so a groundswell of individual decisions about interaction dynamics—perhaps supported by conscious community development—could create community climates that promote newcomer retention.

### 3.5 Implications for cognitive science

Although we specifically examined FOSS communities as our sample of interest, the current focused on these communities as an opportunity to test the dynamical systems view of language and interaction in a real‐world setting and within a naturally occurring dataset (Paxton et al., 2016; Rączaszek‐Leonardi & Kelso, 2008). We hypothesized that the goals of the interaction, the role of the individual within the community, and the culture of the community itself would uniquely shape language use. In other words, we sought out signatures of top‐down and bottom‐up social and cognitive pressures on linguistic behaviors.

Consistent with these hypotheses, we found signatures of time‐, context‐, and goal‐varying behaviors. Not only did a person's status within a community and their goals change their eventual linguistic behaviors, but those behaviors varied across communities and as those communities developed. There was no “one‐size‐fits‐all” language pattern: Language emerged from specific people trying to achieve specific goals within specific contexts.

We further discovered that these linguistic behaviors uniquely impacted critical outcomes. Using the ability to attract and retain a new contributor as a highly salient real‐world outcome of language use, we found that different signatures of language were associated with success in different settings. This again rejects the notion of a static approach to language use or functionality. Both the appearance and the outcome of a specific linguistic behavior, then, were emergent properties—even in high‐stakes, real‐world settings.

In addition to supporting the dynamical systems approach to human behavior, cognition, and interaction (e.g., van Gelder, 1998), the present work has implications for those studying community dynamics in cognitive science and related fields. Psychologists and cognitive scientists have increasingly looked to naturally occurring data as an opportunity to bring theories out of the lab (Goldstone & Lupyan, 2016). Our work demonstrates how this approach can be deployed not only to expand the development of basic science—by testing a specific theory (here, DST) within a specific set of behaviors (here, e.g., engagement, community development, social‐emotional dynamics)—but also to simultaneously give back to communities under examination (here, FOSS contributors).

### 3.6 Limitations and future directions

The present work, like any scientific work, has its own limitations that present future directions. First, we chose to focus here on the scientific Python community; future work should explore whether these dynamics may be specific to the scientific Python community (e.g., by comparing projects from other programming languages) or to essential digital infrastructure (e.g., by comparing projects from smaller communities of users). To complement our focus on newcomer retention, future work should examine the qualitative and quantitative experience of burnout to complete the lifecycle of FOSS contributor activity (Mäntylä et al., 2016), since FOSS communities must also retain long‐term contributors to remain healthy.

Second, we see the present work as fitting with previous research that has focused on the social, emotional, and cognitive aspects of software development and FOSS maintenance (Algoe, 2012; Dabbish et al., 2012; Emmons & Shelton, 2002; França et al., 2014; Steinmacher et al., 2015; Yoshimura & Berzins, 2017). However, we understand that social and community dynamics do not solely shape FOSS projects' ultimate success or failure. For example, previous ethnographic work has identified numerous technical and skill‐based barriers even to the success of an individual contributor (Steinmacher, Silva et al., 2014); at the scale of a FOSS project, there are even more potential impacts, from departures of core maintainers to bugs in project dependencies. By choosing to study only a subset of high‐impact projects for the current work, we have attempted to keep the overall success or failure of a project constant across our sample, but future quantitative work should seek to mirror qualitative work in integrating the social and technical components of software systems (e.g., Steinmacher, Silva et al., 2014) to understand their interplay at scale.

Finally, we adopted a quantitative approach in our analyses to embrace the scale of the naturally occurring data that we used, but we recognize that there is often important nuance lost by taking such a wide‐scale lens. Therefore, the quantitative insights that we provide should also be complemented by future in‐depth qualitative studies of the FOSS community (Howard & Irani, 2019). Through the trace data examined in the current study, we are unable to say *why* these dynamics exist or *how* they emerged. We are unable to address, for example, important but nuanced linguistic devices (e.g., sarcasm, understatement) on community dynamics that could shed light on community supportiveness or toxicity. The current work is a quantitative effort to test theories developed in ethnography and psychology in naturalistic and at‐scale settings, and we hope that our insights will spark new qualitative work to dig deeper into the dynamics we uncovered here. Ultimately, the complexity of the social, emotional, and cognitive processes in these settings is best unraveled through close collaboration between quantitative and qualitative processes (Paxton & Griffiths, 2017).

### 3.7 Conclusion

FOSS has proliferated as the critical international infrastructure, and their reliance on largely volunteer contributors—one of their key strengths—makes the retention of community members vital to their existence. Building on prior work in ethnography and psychology by analyzing community dynamics of FOSS communities on GitHub, we here demonstrated that communication context (i.e., the goals and setting in which a communicative act occurs) and community membership (i.e., being a member or a non‐member of a particular community) shapes the way that people use emotion language and expressions of gratitude. At the same time, FOSS communities exhibited their own dynamics, suggesting that these language dynamics emerge from a fundamental connection to a specific community rather than a common reflection of all FOSS work. Taken together, these findings support the dynamical‐systems view of language (Demjén, 2018; Gibbs & Van Orden, 2012; Paxton et al., 2016) in a naturally occurring dataset by showing the interacting impacts of top‐down and bottom‐up dynamics on functional outcomes: Each contributor's language emerges from the unique pressures of their role in the community and their communicative goals.

### Open Research Badges

This article has earned Open Data and Open Materials badges. Data are available at https://github.com/a‐paxton/oss‐community‐health and materials are available at https://osf.io/6ncwt/.

## Supporting information

Supplementary Table 1. Results of analyses predicting sentiment score with activity and membership.
Supplementary Table 2. Results of analyses predicting log of gratitude count with activity (posted issue [post:issue], comment on an issue [comment:issue], posted pull request [post:PR], or comment on a pull request [comment:PR]) and membership (member or non‐member at time of post).
Supplementary Table 3. Results of analyses of changes in sentiment over time by project, activity (posted issue [post:issue], comment on an issue [comment:issue], posted pull request [post:PR], or comment on a pull request [comment:PR]), and membership (member or nonmember at time of post).
Supplementary Table 4. Results of analyses of changes in log of gratitude count over time by project, activity (posted issue [post:issue], comment on an issue [comment:issue], posted pull request [post:PR], or comment on a pull request [comment:PR]), and membership (member or non‐member at time of post).
Supplementary Table 5. Results of analyses predicting newcomer retention by contribution type and metrics of the community's response to the newcomer
Supplementary Table 6. Results of analyses predicting newcomer retention by contribution type and metrics of the community's response to the newcomer, with two post hoc additions.
Supplementary Table 7. Summary statistics for the current dataset, broken down by project.
Supplementary Figure 1. Histogram of number of projects to which a single user contributes

## Notes

1 Also known as “participatory science” or “citizen science.”

2 We here use sentiment and emotion interchangeably, given their close link. Sentiment is a disposition or emotional position of a person toward a particular target (Broad, 1954). For more on the relations of sentiment, emotion, and other related concepts—especially in computational analyses—see Munzero et al. (2014).

3 However, we also included stability analyses to ensure that the effects were not driven by an arbitrary cutoff; see the Method section for more.

4 We conducted robustness analyses with 7‐ and 10‐contribution cutoffs to ensure that the patterns of results were not due solely to the choice of a 5‐contribution cutoff. Although we observed a small proportion of changes in statistical significance of the results, the direction of all effects remained the same. We expect that the differences in significant results may be due to the decrease in statistical power in the “member” category as the membership cutoff increases. This expectation is bolstered by our finding that there were more changes in the statistically significant results in the 10‐contribution analysis than in the 7‐contribution analysis (compared with the 5‐contribution analysis). All code and results for the robustness analyses are provided in the GitHub and OSF repositories for our project.
