# Supplementary material for: Community, Time, and (Con)text: A Dynamical Systems Analysis of Online Communication and Community Health among Open‐Source Software Communities
Source: Cogn Sci. 2022 May 17;46(5):e13134. doi: 10.1111/cogs.13134 (PMC9287033; doi:10.1111/cogs.13134)
Supplement: Supplementary file 1 — Supplementary Table 1. Results of analyses predicting sentiment score with activity and membership. Supplementary Table 2. Results of analyses predicting log of gratitude count with activity (posted issue [post:issue], comment on an issue [comment:issue], posted pull request [post:PR], or comment on a pull request [comment:PR]) and membership (member or non‐member at time of post). Supplementary Table 3. Results of analyses of changes in sentiment over time by project, activity (posted issue [post:issue], comment on an issue [comment:issue], posted pull request [post:PR], or comment on a pull request [comment:PR]), and membership (member or nonmember at time of post). Supplementary Table 4. Results of analyses of changes in log of gratitude count over time by project, activity (posted issue [post:issue], comment on an issue [comment:issue], posted pull request [post:PR], or comment on a pull request [comment:PR]), and membership (member or non‐member at time of post). Supplementary Table 5. Results of analyses predicting newcomer retention by contribution type and metrics of the community's response to the newcomer Supplementary Table 6. Results of analyses predicting newcomer retention by contribution type and metrics of the community's response to the newcomer, with two post hoc additions. Supplementary Table 7. Summary statistics for the current dataset, broken down by project. Supplementary Figure 1. Histogram of number of projects to which a single user contributes [file COGS-46-0-s001.pdf]

Supplementary Figures

		t-stat	p-val.	p-val adj.	sig.
Main	$\mu_{\text{member}} = \mu_{\text{nonmember}}$	-0.115	0.908	0.942	
	$\mu_{\text{post:issue}} = \mu_{\text{comment:issue}}$	-9.671	0.000	0.000	***
	$\mu_{\text{post:PR}} = \mu_{\text{comment:PR}}$	-12.528	0.000	0.000	***
	$\mu_{\text{post:issue}} = \mu_{\text{post:PR}}$	-0.692	0.489	0.594	
	$\mu_{\text{comment:issue}} = \mu_{\text{comment:PR}}$	-3.660	0.000	0.001	***
2W	$\mu_{\text{post:issue}\times\text{member}} = \mu_{\text{post:issue}\times\text{nonmember}}$	-0.768	0.442	0.549	
	$\mu_{\text{comment:issue}\times\text{member}} = \mu_{\text{comment:issue}\times\text{nonmember}}$	-2.489	0.013	0.024	*
	$\mu_{\text{post:PR}\times\text{member}} = \mu_{\text{post:PR}\times\text{nonmember}}$	-2.791	0.005	0.010	*
	$\mu_{\text{comment:PR}\times\text{member}} = \mu_{\text{comment:PR}\times\text{nonmember}}$	-0.561	0.575	0.674	
	$\mu_{\text{post:issue}\times\text{member}} = \mu_{\text{comment:issue}\times\text{member}}$	-7.957	0.000	0.000	***
	$\mu_{\text{post:issue}\times\text{nonmember}} = \mu_{\text{comment:issue}\times\text{nonmember}}$	-10.126	0.000	0.000	***
	$\mu_{\text{post:PR}\times\text{member}} = \mu_{\text{comment:PR}\times\text{member}}$	-11.901	0.000	0.000	***
	$\mu_{\text{post:PR}\times\text{nonmember}} = \mu_{\text{comment:PR}\times\text{nonmember}}$	-9.623	0.000	0.000	***
	$\mu_{\text{post:issue}\times\text{member}} = \mu_{\text{post:PR}\times\text{member}}$	0.232	0.816	0.884	
	$\mu_{\text{post:issue}\times\text{nonmember}} = \mu_{\text{post:PR}\times\text{nonmember}}$	-1.828	0.068	0.105	
	$\mu_{\text{comment:issue}\times\text{member}} = \mu_{\text{comment:PR}\times\text{member}}$	-3.625	0.000	0.001	***
	$\mu_{\text{comment:issue}\times\text{nonmember}} = \mu_{\text{comment:PR}\times\text{nonmember}}$	-1.797	0.072	0.110	
scikit-learn	$\mu_{\text{post:issue}\times\text{member}} = \mu_{\text{post:issue}\times\text{nonmember}}$	-0.712	0.477	0.585	
	$\mu_{\text{comment:issue}\times\text{member}} = \mu_{\text{comment:issue}\times\text{nonmember}}$	-3.612	0.000	0.001	***
	$\mu_{\text{post:PR}\times\text{member}} = \mu_{\text{post:PR}\times\text{nonmember}}$	-0.011	0.991	0.993	
	$\mu_{\text{comment:PR}\times\text{member}} = \mu_{\text{comment:PR}\times\text{nonmember}}$	-0.842	0.400	0.502	
	$\mu_{\text{post:issue}\times\text{member}} = \mu_{\text{comment:issue}\times\text{member}}$	-8.913	0.000	0.000	***
	$\mu_{\text{post:issue}\times\text{nonmember}} = \mu_{\text{comment:issue}\times\text{nonmember}}$	-12.536	0.000	0.000	***
	$\mu_{\text{post:PR}\times\text{member}} = \mu_{\text{comment:PR}\times\text{member}}$	-12.627	0.000	0.000	***
	$\mu_{\text{post:PR}\times\text{nonmember}} = \mu_{\text{comment:PR}\times\text{nonmember}}$	-13.351	0.000	0.000	***
	$\mu_{\text{post:issue}\times\text{member}} = \mu_{\text{post:PR}\times\text{member}}$	-1.877	0.061	0.095	.
	$\mu_{\text{post:issue}\times\text{nonmember}} = \mu_{\text{post:PR}\times\text{nonmember}}$	-1.344	0.179	0.250	

Continued on next page

		t-stat	p-val.	p-val adj.	sig.
	$\mu_{\text{comment:issue×member}} = \mu_{\text{comment:PR×member}}$	-6.375	0.000	0.000	***
	$\mu_{\text{comment:issue×nonmember}} = \mu_{\text{comment:PR×nonmember}}$	-3.797	0.000	0.000	***
scikit-image	$\mu_{\text{post:issue×member}} = \mu_{\text{post:issue×nonmember}}$	-1.315	0.189	0.257	
	$\mu_{\text{comment:issue×member}} = \mu_{\text{comment:issue×nonmember}}$	-1.323	0.186	0.256	
	$\mu_{\text{post:PR×member}} = \mu_{\text{post:PR×nonmember}}$	-7.070	0.000	0.000	***
	$\mu_{\text{comment:PR×member}} = \mu_{\text{comment:PR×nonmember}}$	-0.009	0.993	0.993	
	$\mu_{\text{post:issue×member}} = \mu_{\text{comment:issue×member}}$	-7.624	0.000	0.000	***
	$\mu_{\text{post:issue×nonmember}} = \mu_{\text{comment:issue×nonmember}}$	-7.252	0.000	0.000	***
	$\mu_{\text{post:PR×member}} = \mu_{\text{comment:PR×member}}$	-8.901	0.000	0.000	***
	$\mu_{\text{post:PR×nonmember}} = \mu_{\text{comment:PR×nonmember}}$	0.626	0.532	0.639	
	$\mu_{\text{post:issue×member}} = \mu_{\text{post:PR×member}}$	-3.255	0.001	0.002	**
	$\mu_{\text{post:issue×nonmember}} = \mu_{\text{post:PR×nonmember}}$	-7.981	0.000	0.000	***
	$\mu_{\text{comment:issue×member}} = \mu_{\text{comment:PR×member}}$	-4.003	0.000	0.000	***
	$\mu_{\text{comment:issue×nonmember}} = \mu_{\text{comment:PR×nonmember}}$	-2.137	0.033	0.056	.
matplotlib	$\mu_{\text{post:issue×member}} = \mu_{\text{post:issue×nonmember}}$	-0.933	0.351	0.449	
	$\mu_{\text{comment:issue×member}} = \mu_{\text{comment:issue×nonmember}}$	-1.965	0.049	0.080	.
	$\mu_{\text{post:PR×member}} = \mu_{\text{post:PR×nonmember}}$	-0.928	0.353	0.449	
	$\mu_{\text{comment:PR×member}} = \mu_{\text{comment:PR×nonmember}}$	-1.746	0.081	0.122	
	$\mu_{\text{post:issue×member}} = \mu_{\text{comment:issue×member}}$	-7.369	0.000	0.000	***
	$\mu_{\text{post:issue×nonmember}} = \mu_{\text{comment:issue×nonmember}}$	-9.991	0.000	0.000	***
	$\mu_{\text{post:PR×member}} = \mu_{\text{comment:PR×member}}$	-11.454	0.000	0.000	***
	$\mu_{\text{post:PR×nonmember}} = \mu_{\text{comment:PR×nonmember}}$	-8.945	0.000	0.000	***
	$\mu_{\text{post:issue×member}} = \mu_{\text{post:PR×member}}$	-0.556	0.578	0.674	
	$\mu_{\text{post:issue×nonmember}} = \mu_{\text{post:PR×nonmember}}$	-0.517	0.605	0.691	
	$\mu_{\text{comment:issue×member}} = \mu_{\text{comment:PR×member}}$	-2.933	0.003	0.007	**
	$\mu_{\text{comment:issue×nonmember}} = \mu_{\text{comment:PR×nonmember}}$	-2.713	0.007	0.013	*
	$\mu_{\text{post:issue×member}} = \mu_{\text{post:issue×nonmember}}$	-1.568	0.117	0.169	

Continued on next page

		t-stat	p-val.	p-val adj.	sig.
	$\mu_{\text{comment:issue}\times\text{member}} = \mu_{\text{comment:issue}\times\text{nonmember}}$	-1.406	0.160	0.229	
	$\mu_{\text{post:PR}\times\text{member}} = \mu_{\text{post:PR}\times\text{nonmember}}$	-0.520	0.603	0.691	
	$\mu_{\text{comment:PR}\times\text{member}} = \mu_{\text{comment:PR}\times\text{nonmember}}$	-2.075	0.038	0.063	.
	$\mu_{\text{post:issue}\times\text{member}} = \mu_{\text{comment:issue}\times\text{member}}$	-1.882	0.060	0.095	.
	$\mu_{\text{post:issue}\times\text{nonmember}} = \mu_{\text{comment:issue}\times\text{nonmember}}$	-2.890	0.004	0.008	**
	$\mu_{\text{post:PR}\times\text{member}} = \mu_{\text{comment:PR}\times\text{member}}$	-2.279	0.023	0.041	*
	$\mu_{\text{post:PR}\times\text{nonmember}} = \mu_{\text{comment:PR}\times\text{nonmember}}$	-3.404	0.001	0.001	**
	$\mu_{\text{post:issue}\times\text{member}} = \mu_{\text{post:PR}\times\text{member}}$	-0.069	0.945	0.962	
	$\mu_{\text{post:issue}\times\text{nonmember}} = \mu_{\text{post:PR}\times\text{nonmember}}$	1.071	0.284	0.378	
	$\mu_{\text{comment:issue}\times\text{member}} = \mu_{\text{comment:PR}\times\text{member}}$	-0.193	0.847	0.903	
	$\mu_{\text{comment:issue}\times\text{nonmember}} = \mu_{\text{comment:PR}\times\text{nonmember}}$	-1.570	0.116	0.169	
pandas	$\mu_{\text{post:issue}\times\text{member}} = \mu_{\text{post:issue}\times\text{nonmember}}$	-1.052	0.293	0.385	
	$\mu_{\text{comment:issue}\times\text{member}} = \mu_{\text{comment:issue}\times\text{nonmember}}$	-5.483	0.000	0.000	***
	$\mu_{\text{post:PR}\times\text{member}} = \mu_{\text{post:PR}\times\text{nonmember}}$	-4.700	0.000	0.000	***
	$\mu_{\text{comment:PR}\times\text{member}} = \mu_{\text{comment:PR}\times\text{nonmember}}$	1.397	0.162	0.229	
	$\mu_{\text{post:issue}\times\text{member}} = \mu_{\text{comment:issue}\times\text{member}}$	-9.886	0.000	0.000	***
	$\mu_{\text{post:issue}\times\text{nonmember}} = \mu_{\text{comment:issue}\times\text{nonmember}}$	-15.061	0.000	0.000	***
	$\mu_{\text{post:PR}\times\text{member}} = \mu_{\text{comment:PR}\times\text{member}}$	-18.351	0.000	0.000	***
	$\mu_{\text{post:PR}\times\text{nonmember}} = \mu_{\text{comment:PR}\times\text{nonmember}}$	-9.050	0.000	0.000	***
	$\mu_{\text{post:issue}\times\text{member}} = \mu_{\text{post:PR}\times\text{member}}$	4.587	0.000	0.000	***
	$\mu_{\text{post:issue}\times\text{nonmember}} = \mu_{\text{post:PR}\times\text{nonmember}}$	0.314	0.754	0.835	
	$\mu_{\text{comment:issue}\times\text{member}} = \mu_{\text{comment:PR}\times\text{member}}$	-3.839	0.000	0.000	***
	$\mu_{\text{comment:issue}\times\text{nonmember}} = \mu_{\text{comment:PR}\times\text{nonmember}}$	2.691	0.007	0.014	*
	$\mu_{\text{post:issue}\times\text{member}} = \mu_{\text{post:issue}\times\text{nonmember}}$	-3.035	0.002	0.005	**
	$\mu_{\text{comment:issue}\times\text{member}} = \mu_{\text{comment:issue}\times\text{nonmember}}$	-5.669	0.000	0.000	***
	$\mu_{\text{post:PR}\times\text{member}} = \mu_{\text{post:PR}\times\text{nonmember}}$	-3.921	0.000	0.000	***
	$\mu_{\text{comment:PR}\times\text{member}} = \mu_{\text{comment:PR}\times\text{nonmember}}$	-2.119	0.034	0.057	.

Continued on next page

scipy

		t-stat	p-val.	p-val adj.	sig.
	$\mu_{post:issue \times member} = \mu_{comment:issue \times member}$	-8.790	0.000	0.000	***
	$\mu_{post:issue \times nonmember} = \mu_{comment:issue \times nonmember}$	-11.828	0.000	0.000	***
	$\mu_{post:PR \times member} = \mu_{comment:PR \times member}$	-16.597	0.000	0.000	***
	$\mu_{post:PR \times nonmember} = \mu_{comment:PR \times nonmember}$	-10.357	0.000	0.000	***
	$\mu_{post:issue \times member} = \mu_{post:PR \times member}$	-2.263	0.024	0.042	*
	$\mu_{post:issue \times nonmember} = \mu_{post:PR \times nonmember}$	-2.847	0.004	0.009	**
	$\mu_{comment:issue \times member} = \mu_{comment:PR \times member}$	-9.017	0.000	0.000	***
	$\mu_{comment:issue \times nonmember} = \mu_{comment:PR \times nonmember}$	-4.262	0.000	0.000	***
numpy	$\mu_{post:issue \times member} = \mu_{post:issue \times nonmember}$	0.092	0.927	0.952	
	$\mu_{comment:issue \times member} = \mu_{comment:issue \times nonmember}$	-2.542	0.011	0.021	*
	$\mu_{post:PR \times member} = \mu_{post:PR \times nonmember}$	-0.226	0.822	0.884	
	$\mu_{comment:PR \times member} = \mu_{comment:PR \times nonmember}$	0.480	0.631	0.713	
	$\mu_{post:issue \times member} = \mu_{comment:issue \times member}$	-9.899	0.000	0.000	***
	$\mu_{post:issue \times nonmember} = \mu_{comment:issue \times nonmember}$	-13.286	0.000	0.000	***
	$\mu_{post:PR \times member} = \mu_{comment:PR \times member}$	-16.979	0.000	0.000	***
	$\mu_{post:PR \times nonmember} = \mu_{comment:PR \times nonmember}$	-11.678	0.000	0.000	***
	$\mu_{post:issue \times member} = \mu_{post:PR \times member}$	-1.996	0.046	0.075	.
	$\mu_{post:issue \times nonmember} = \mu_{post:PR \times nonmember}$	-2.191	0.028	0.049	*
	$\mu_{comment:issue \times member} = \mu_{comment:PR \times member}$	-9.492	0.000	0.000	***
	$\mu_{comment:issue \times nonmember} = \mu_{comment:PR \times nonmember}$	-5.002	0.000	0.000	***
sphinx-gallery	$\mu_{post:issue \times member} = \mu_{post:issue \times nonmember}$	0.570	0.568	0.674	
	$\mu_{comment:issue \times member} = \mu_{comment:issue \times nonmember}$	-0.354	0.723	0.809	
	$\mu_{post:PR \times member} = \mu_{post:PR \times nonmember}$	-1.625	0.104	0.155	
	$\mu_{comment:PR \times member} = \mu_{comment:PR \times nonmember}$	0.135	0.892	0.934	
	$\mu_{post:issue \times member} = \mu_{comment:issue \times member}$	-3.577	0.000	0.001	***
	$\mu_{post:issue \times nonmember} = \mu_{comment:issue \times nonmember}$	-4.137	0.000	0.000	***
	$\mu_{post:PR \times member} = \mu_{comment:PR \times member}$	-3.462	0.001	0.001	**

Continued on next page

	t-stat	p-val.	p-val adj.	sig.
$\mu_{\text{post:PR}\times\text{nonmember}} = \mu_{\text{comment:PR}\times\text{nonmember}}$	-0.182	0.856	0.904	
$\mu_{\text{post:issue}\times\text{member}} = \mu_{\text{post:PR}\times\text{member}}$	-0.298	0.766	0.840	
$\mu_{\text{post:issue}\times\text{nonmember}} = \mu_{\text{post:PR}\times\text{nonmember}}$	-2.216	0.027	0.047	*
$\mu_{\text{comment:issue}\times\text{member}} = \mu_{\text{comment:PR}\times\text{member}}$	1.224	0.221	0.297	
$\mu_{\text{comment:issue}\times\text{nonmember}} = \mu_{\text{comment:PR}\times\text{nonmember}}$	1.034	0.301	0.391	

Supplementary Table 1

*Results of analyses predicting sentiment score with activity and membership. All p-values adjusted for multiple comparisons using Benjamini-Hochberg. Legend: . = p < .10; * = p < .05; ** = p < .001; *** = p < .0001*

		t-stat	p-val.	p-val adj.	sig.
Main	$\mu_{\text{member}} = \mu_{\text{nonmember}}$	-3.383	0.001	0.001	**
	$\mu_{\text{post:issue}} = \mu_{\text{comment:issue}}$	-1.619	0.105	0.135	
	$\mu_{\text{post:PR}} = \mu_{\text{comment:PR}}$	-6.710	0.000	0.000	***
	$\mu_{\text{post:issue}} = \mu_{\text{post:PR}}$	2.418	0.016	0.022	*
	$\mu_{\text{comment:issue}} = \mu_{\text{comment:PR}}$	-2.671	0.008	0.011	*
2W	$\mu_{\text{post:issue}\times\text{member}} = \mu_{\text{post:issue}\times\text{nonmember}}$	-5.139	0.000	0.000	***
	$\mu_{\text{comment:issue}\times\text{member}} = \mu_{\text{comment:issue}\times\text{nonmember}}$	-8.616	0.000	0.000	***
	$\mu_{\text{post:PR}\times\text{member}} = \mu_{\text{post:PR}\times\text{nonmember}}$	-0.874	0.382	0.441	
	$\mu_{\text{comment:PR}\times\text{member}} = \mu_{\text{comment:PR}\times\text{nonmember}}$	-1.084	0.278	0.328	
	$\mu_{\text{post:issue}\times\text{member}} = \mu_{\text{comment:issue}\times\text{member}}$	-2.752	0.006	0.009	**
	$\mu_{\text{post:issue}\times\text{nonmember}} = \mu_{\text{comment:issue}\times\text{nonmember}}$	-6.083	0.000	0.000	***
	$\mu_{\text{post:PR}\times\text{member}} = \mu_{\text{comment:PR}\times\text{member}}$	-8.378	0.000	0.000	***
	$\mu_{\text{post:PR}\times\text{nonmember}} = \mu_{\text{comment:PR}\times\text{nonmember}}$	-8.442	0.000	0.000	***
	$\mu_{\text{post:issue}\times\text{member}} = \mu_{\text{post:PR}\times\text{member}}$	0.375	0.708	0.762	
	$\mu_{\text{post:issue}\times\text{nonmember}} = \mu_{\text{post:PR}\times\text{nonmember}}$	4.622	0.000	0.000	***
	$\mu_{\text{comment:issue}\times\text{member}} = \mu_{\text{comment:PR}\times\text{member}}$	-5.263	0.000	0.000	***
	$\mu_{\text{comment:issue}\times\text{nonmember}} = \mu_{\text{comment:PR}\times\text{nonmember}}$	2.278	0.023	0.031	*
scikit-learn	$\mu_{\text{post:issue}\times\text{member}} = \mu_{\text{post:issue}\times\text{nonmember}}$	-14.666	0.000	0.000	***
	$\mu_{\text{comment:issue}\times\text{member}} = \mu_{\text{comment:issue}\times\text{nonmember}}$	-28.530	0.000	0.000	***
	$\mu_{\text{post:PR}\times\text{member}} = \mu_{\text{post:PR}\times\text{nonmember}}$	-1.397	0.162	0.202	
	$\mu_{\text{comment:PR}\times\text{member}} = \mu_{\text{comment:PR}\times\text{nonmember}}$	-10.268	0.000	0.000	***
	$\mu_{\text{post:issue}\times\text{member}} = \mu_{\text{comment:issue}\times\text{member}}$	-9.181	0.000	0.000	***
	$\mu_{\text{post:issue}\times\text{nonmember}} = \mu_{\text{comment:issue}\times\text{nonmember}}$	-5.942	0.000	0.000	***
	$\mu_{\text{post:PR}\times\text{member}} = \mu_{\text{comment:PR}\times\text{member}}$	-22.696	0.000	0.000	***
	$\mu_{\text{post:PR}\times\text{nonmember}} = \mu_{\text{comment:PR}\times\text{nonmember}}$	-22.459	0.000	0.000	***
	$\mu_{\text{post:issue}\times\text{member}} = \mu_{\text{post:PR}\times\text{member}}$	-1.162	0.245	0.292	
	$\mu_{\text{post:issue}\times\text{nonmember}} = \mu_{\text{post:PR}\times\text{nonmember}}$	13.215	0.000	0.000	***

Continued on next page

		t-stat	p-val.	p-val adj.	sig.
	$\mu_{\text{comment:issue}\times\text{member}} = \mu_{\text{comment:PR}\times\text{member}}$	-31.005	0.000	0.000	***
	$\mu_{\text{comment:issue}\times\text{nonmember}} = \mu_{\text{comment:PR}\times\text{nonmember}}$	-0.071	0.944	0.952	
scikit-image	$\mu_{\text{post:issue}\times\text{member}} = \mu_{\text{post:issue}\times\text{nonmember}}$	-5.820	0.000	0.000	***
	$\mu_{\text{comment:issue}\times\text{member}} = \mu_{\text{comment:issue}\times\text{nonmember}}$	-10.310	0.000	0.000	***
	$\mu_{\text{post:PR}\times\text{member}} = \mu_{\text{post:PR}\times\text{nonmember}}$	-1.363	0.173	0.212	
	$\mu_{\text{comment:PR}\times\text{member}} = \mu_{\text{comment:PR}\times\text{nonmember}}$	-0.626	0.531	0.589	
	$\mu_{\text{post:issue}\times\text{member}} = \mu_{\text{comment:issue}\times\text{member}}$	-5.881	0.000	0.000	***
	$\mu_{\text{post:issue}\times\text{nonmember}} = \mu_{\text{comment:issue}\times\text{nonmember}}$	-4.413	0.000	0.000	***
	$\mu_{\text{post:PR}\times\text{member}} = \mu_{\text{comment:PR}\times\text{member}}$	-18.886	0.000	0.000	***
	$\mu_{\text{post:PR}\times\text{nonmember}} = \mu_{\text{comment:PR}\times\text{nonmember}}$	-10.768	0.000	0.000	***
	$\mu_{\text{post:issue}\times\text{member}} = \mu_{\text{post:PR}\times\text{member}}$	-0.247	0.805	0.842	
	$\mu_{\text{post:issue}\times\text{nonmember}} = \mu_{\text{post:PR}\times\text{nonmember}}$	4.725	0.000	0.000	***
	$\mu_{\text{comment:issue}\times\text{member}} = \mu_{\text{comment:PR}\times\text{member}}$	-16.860	0.000	0.000	***
	$\mu_{\text{comment:issue}\times\text{nonmember}} = \mu_{\text{comment:PR}\times\text{nonmember}}$	-0.153	0.878	0.902	
matplotlib	$\mu_{\text{post:issue}\times\text{member}} = \mu_{\text{post:issue}\times\text{nonmember}}$	-5.112	0.000	0.000	***
	$\mu_{\text{comment:issue}\times\text{member}} = \mu_{\text{comment:issue}\times\text{nonmember}}$	-35.211	0.000	0.000	***
	$\mu_{\text{post:PR}\times\text{member}} = \mu_{\text{post:PR}\times\text{nonmember}}$	-1.525	0.127	0.161	
	$\mu_{\text{comment:PR}\times\text{member}} = \mu_{\text{comment:PR}\times\text{nonmember}}$	-17.307	0.000	0.000	***
	$\mu_{\text{post:issue}\times\text{member}} = \mu_{\text{comment:issue}\times\text{member}}$	-0.268	0.789	0.833	
	$\mu_{\text{post:issue}\times\text{nonmember}} = \mu_{\text{comment:issue}\times\text{nonmember}}$	-15.149	0.000	0.000	***
	$\mu_{\text{post:PR}\times\text{member}} = \mu_{\text{comment:PR}\times\text{member}}$	-16.944	0.000	0.000	***
	$\mu_{\text{post:PR}\times\text{nonmember}} = \mu_{\text{comment:PR}\times\text{nonmember}}$	-16.938	0.000	0.000	***
	$\mu_{\text{post:issue}\times\text{member}} = \mu_{\text{post:PR}\times\text{member}}$	3.528	0.000	0.001	***
	$\mu_{\text{post:issue}\times\text{nonmember}} = \mu_{\text{post:PR}\times\text{nonmember}}$	6.776	0.000	0.000	***
	$\mu_{\text{comment:issue}\times\text{member}} = \mu_{\text{comment:PR}\times\text{member}}$	-16.203	0.000	0.000	***
	$\mu_{\text{comment:issue}\times\text{nonmember}} = \mu_{\text{comment:PR}\times\text{nonmember}}$	2.659	0.008	0.011	*
	$\mu_{\text{post:issue}\times\text{member}} = \mu_{\text{post:issue}\times\text{nonmember}}$	-5.731	0.000	0.000	***

Continued on next page

		t-stat	p-val.	p-val adj.	sig.
	$\mu_{\text{comment:issue}\times\text{member}} = \mu_{\text{comment:issue}\times\text{nonmember}}$	-6.625	0.000	0.000	***
	$\mu_{\text{post:PR}\times\text{member}} = \mu_{\text{post:PR}\times\text{nonmember}}$	0.014	0.989	0.989	
	$\mu_{\text{comment:PR}\times\text{member}} = \mu_{\text{comment:PR}\times\text{nonmember}}$	5.396	0.000	0.000	***
	$\mu_{\text{post:issue}\times\text{member}} = \mu_{\text{comment:issue}\times\text{member}}$	-3.359	0.001	0.001	**
	$\mu_{\text{post:issue}\times\text{nonmember}} = \mu_{\text{comment:issue}\times\text{nonmember}}$	-0.672	0.502	0.561	
	$\mu_{\text{post:PR}\times\text{member}} = \mu_{\text{comment:PR}\times\text{member}}$	-10.993	0.000	0.000	***
	$\mu_{\text{post:PR}\times\text{nonmember}} = \mu_{\text{comment:PR}\times\text{nonmember}}$	-3.794	0.000	0.000	***
	$\mu_{\text{post:issue}\times\text{member}} = \mu_{\text{post:PR}\times\text{member}}$	-0.836	0.403	0.460	
	$\mu_{\text{post:issue}\times\text{nonmember}} = \mu_{\text{post:PR}\times\text{nonmember}}$	4.967	0.000	0.000	***
	$\mu_{\text{comment:issue}\times\text{member}} = \mu_{\text{comment:PR}\times\text{member}}$	-10.532	0.000	0.000	***
	$\mu_{\text{comment:issue}\times\text{nonmember}} = \mu_{\text{comment:PR}\times\text{nonmember}}$	1.947	0.052	0.069	.
pandas	$\mu_{\text{post:issue}\times\text{member}} = \mu_{\text{post:issue}\times\text{nonmember}}$	-11.251	0.000	0.000	***
	$\mu_{\text{comment:issue}\times\text{member}} = \mu_{\text{comment:issue}\times\text{nonmember}}$	-44.771	0.000	0.000	***
	$\mu_{\text{post:PR}\times\text{member}} = \mu_{\text{post:PR}\times\text{nonmember}}$	-2.763	0.006	0.009	**
	$\mu_{\text{comment:PR}\times\text{member}} = \mu_{\text{comment:PR}\times\text{nonmember}}$	9.658	0.000	0.000	***
	$\mu_{\text{post:issue}\times\text{member}} = \mu_{\text{comment:issue}\times\text{member}}$	-11.531	0.000	0.000	***
	$\mu_{\text{post:issue}\times\text{nonmember}} = \mu_{\text{comment:issue}\times\text{nonmember}}$	-24.827	0.000	0.000	***
	$\mu_{\text{post:PR}\times\text{member}} = \mu_{\text{comment:PR}\times\text{member}}$	-34.411	0.000	0.000	***
	$\mu_{\text{post:PR}\times\text{nonmember}} = \mu_{\text{comment:PR}\times\text{nonmember}}$	-13.474	0.000	0.000	***
	$\mu_{\text{post:issue}\times\text{member}} = \mu_{\text{post:PR}\times\text{member}}$	1.225	0.220	0.265	
	$\mu_{\text{post:issue}\times\text{nonmember}} = \mu_{\text{post:PR}\times\text{nonmember}}$	7.148	0.000	0.000	***
	$\mu_{\text{comment:issue}\times\text{member}} = \mu_{\text{comment:PR}\times\text{member}}$	-42.608	0.000	0.000	***
	$\mu_{\text{comment:issue}\times\text{nonmember}} = \mu_{\text{comment:PR}\times\text{nonmember}}$	21.295	0.000	0.000	***
	$\mu_{\text{post:issue}\times\text{member}} = \mu_{\text{post:issue}\times\text{nonmember}}$	-7.441	0.000	0.000	***
	$\mu_{\text{comment:issue}\times\text{member}} = \mu_{\text{comment:issue}\times\text{nonmember}}$	-23.611	0.000	0.000	***
	$\mu_{\text{post:PR}\times\text{member}} = \mu_{\text{post:PR}\times\text{nonmember}}$	-2.325	0.020	0.028	*
	$\mu_{\text{comment:PR}\times\text{member}} = \mu_{\text{comment:PR}\times\text{nonmember}}$	-3.495	0.000	0.001	***

Continued on next page

scipy

		t-stat	p-val.	p-val adj.	sig.
	$\mu_{\text{post:issue}\times\text{member}} = \mu_{\text{comment:issue}\times\text{member}}$	-4.636	0.000	0.000	***
	$\mu_{\text{post:issue}\times\text{nonmember}} = \mu_{\text{comment:issue}\times\text{nonmember}}$	-9.932	0.000	0.000	***
	$\mu_{\text{post:PR}\times\text{member}} = \mu_{\text{comment:PR}\times\text{member}}$	-25.039	0.000	0.000	***
	$\mu_{\text{post:PR}\times\text{nonmember}} = \mu_{\text{comment:PR}\times\text{nonmember}}$	-16.238	0.000	0.000	***
	$\mu_{\text{post:issue}\times\text{member}} = \mu_{\text{post:PR}\times\text{member}}$	-0.344	0.731	0.779	
	$\mu_{\text{post:issue}\times\text{nonmember}} = \mu_{\text{post:PR}\times\text{nonmember}}$	6.361	0.000	0.000	***
	$\mu_{\text{comment:issue}\times\text{member}} = \mu_{\text{comment:PR}\times\text{member}}$	-30.817	0.000	0.000	***
	$\mu_{\text{comment:issue}\times\text{nonmember}} = \mu_{\text{comment:PR}\times\text{nonmember}}$	-0.132	0.895	0.911	
numpy	$\mu_{\text{post:issue}\times\text{member}} = \mu_{\text{post:issue}\times\text{nonmember}}$	-5.873	0.000	0.000	***
	$\mu_{\text{comment:issue}\times\text{member}} = \mu_{\text{comment:issue}\times\text{nonmember}}$	-31.858	0.000	0.000	***
	$\mu_{\text{post:PR}\times\text{member}} = \mu_{\text{post:PR}\times\text{nonmember}}$	-1.053	0.292	0.340	
	$\mu_{\text{comment:PR}\times\text{member}} = \mu_{\text{comment:PR}\times\text{nonmember}}$	-5.000	0.000	0.000	***
	$\mu_{\text{post:issue}\times\text{member}} = \mu_{\text{comment:issue}\times\text{member}}$	-2.655	0.008	0.011	*
	$\mu_{\text{post:issue}\times\text{nonmember}} = \mu_{\text{comment:issue}\times\text{nonmember}}$	-14.205	0.000	0.000	***
	$\mu_{\text{post:PR}\times\text{member}} = \mu_{\text{comment:PR}\times\text{member}}$	-24.072	0.000	0.000	***
	$\mu_{\text{post:PR}\times\text{nonmember}} = \mu_{\text{comment:PR}\times\text{nonmember}}$	-15.473	0.000	0.000	***
	$\mu_{\text{post:issue}\times\text{member}} = \mu_{\text{post:PR}\times\text{member}}$	1.763	0.078	0.101	
	$\mu_{\text{post:issue}\times\text{nonmember}} = \mu_{\text{post:PR}\times\text{nonmember}}$	5.998	0.000	0.000	***
	$\mu_{\text{comment:issue}\times\text{member}} = \mu_{\text{comment:PR}\times\text{member}}$	-32.582	0.000	0.000	***
	$\mu_{\text{comment:issue}\times\text{nonmember}} = \mu_{\text{comment:PR}\times\text{nonmember}}$	3.773	0.000	0.000	***
sphinx-gallery	$\mu_{\text{post:issue}\times\text{member}} = \mu_{\text{post:issue}\times\text{nonmember}}$	-1.797	0.072	0.095	.
	$\mu_{\text{comment:issue}\times\text{member}} = \mu_{\text{comment:issue}\times\text{nonmember}}$	-6.165	0.000	0.000	***
	$\mu_{\text{post:PR}\times\text{member}} = \mu_{\text{post:PR}\times\text{nonmember}}$	-0.743	0.457	0.517	
	$\mu_{\text{comment:PR}\times\text{member}} = \mu_{\text{comment:PR}\times\text{nonmember}}$	-0.185	0.853	0.884	
	$\mu_{\text{post:issue}\times\text{member}} = \mu_{\text{comment:issue}\times\text{member}}$	-0.597	0.550	0.604	
	$\mu_{\text{post:issue}\times\text{nonmember}} = \mu_{\text{comment:issue}\times\text{nonmember}}$	-2.025	0.043	0.058	.
	$\mu_{\text{post:PR}\times\text{member}} = \mu_{\text{comment:PR}\times\text{member}}$	-3.992	0.000	0.000	***

Continued on next page

	t-stat	p-val.	p-val adj.	sig.
$\mu_{\text{post:PR}\times\text{nonmember}} = \mu_{\text{comment:PR}\times\text{nonmember}}$	-1.480	0.139	0.174	
$\mu_{\text{post:issue}\times\text{member}} = \mu_{\text{post:PR}\times\text{member}}$	0.518	0.605	0.657	
$\mu_{\text{post:issue}\times\text{nonmember}} = \mu_{\text{post:PR}\times\text{nonmember}}$	1.241	0.215	0.261	
$\mu_{\text{comment:issue}\times\text{member}} = \mu_{\text{comment:PR}\times\text{member}}$	-4.506	0.000	0.000	***
$\mu_{\text{comment:issue}\times\text{nonmember}} = \mu_{\text{comment:PR}\times\text{nonmember}}$	2.577	0.010	0.014	*

Supplementary Table 2

*Results of analyses predicting log of gratitude count with activity (posted issue [`post:issue`], comment on an issue [`comment:issue`], posted pull request [`post:PR`], or comment on a pull request [`comment:PR`]) and membership (`member` or `nonmember` at time of post). The "Main" results include only the main effects of contribution type and membership. The "2W" results include two-way interaction terms among the main terms. The "3W" results include additional interactions with community membership. All p-values adjusted for multiple comparisons using Benjamini-Hochberg. Legend: . = p < .10; * = p < .05; ** = p < .001; *** = p < .0001*

Project	Contrast	Year	t-stat	p-val.	p-val adj.	sig.
matplotlib	comment:PR × member	2011	-2.298	0.022	0.087	.
		2012	-1.525	0.127	0.309	
		2013	-3.082	0.002	0.018	*
		2014	-2.787	0.005	0.037	*
		2015	-5.019	0.000	0.000	***
		2016	-4.489	0.000	0.000	***
		2017	-2.416	0.016	0.073	.
		2018	-1.108	0.268	0.465	
matplotlib	comment:PR × nonmember	2011	-1.037	0.300	0.501	
		2012	1.347	0.178	0.364	
		2013	0.167	0.868	0.936	
		2014	0.179	0.858	0.934	
		2015	-1.699	0.089	0.244	
		2016	-2.853	0.004	0.033	*
		2017	-2.803	0.005	0.036	*
		2018	-0.388	0.698	0.859	
matplotlib	comment:issue × member	2011	-4.037	0.000	0.001	***
		2012	-0.787	0.431	0.649	
		2013	-1.018	0.309	0.510	
		2014	-1.866	0.062	0.190	
		2015	-0.823	0.410	0.631	
		2016	1.177	0.239	0.445	
		2017	-1.467	0.142	0.324	
		2018	0.232	0.817	0.918	
matplotlib	comment:issue × nonmember	2011	0.080	0.936	0.960	
		2012	0.105	0.916	0.959	
		2013	-0.904	0.366	0.588	

Continued on next page

Project	Contrast	Year	t-stat	p-val.	p-val adj.	sig.
		2014	-3.460	0.001	0.006	**
		2015	-1.188	0.235	0.438	
		2016	-0.247	0.805	0.909	
		2017	-1.737	0.082	0.235	
		2018	-1.692	0.091	0.244	
matplotlib	post:PR × member	2011	-4.584	0.000	0.000	***
		2012	1.706	0.088	0.242	
		2013	-0.406	0.685	0.851	
		2014	0.400	0.689	0.851	
		2015	-0.114	0.909	0.959	
		2016	1.778	0.075	0.223	
		2017	1.933	0.053	0.168	
		2018	0.721	0.471	0.692	
matplotlib	post:PR × nonmember	2011	-1.594	0.111	0.283	
		2012	0.100	0.920	0.959	
		2013	-1.412	0.158	0.340	
		2014	1.324	0.185	0.371	
		2015	-0.063	0.950	0.970	
		2016	-0.663	0.508	0.723	
		2017	-0.623	0.534	0.748	
		2018	-1.625	0.104	0.272	
matplotlib	post:issue × member	2011	2.097	0.036	0.131	
		2012	-2.359	0.018	0.081	.
		2013	-3.170	0.002	0.015	*
		2014	-0.077	0.938	0.960	
		2015	2.562	0.010	0.054	.
		2016	1.964	0.050	0.165	

Continued on next page

Project	Contrast	Year	t-stat	p-val.	p-val adj.	sig.
		2017	-1.435	0.151	0.334	
		2018	-2.649	0.008	0.047	*
matplotlib	post:issue × nonmember	2011	-1.233	0.218	0.419	
		2012	1.418	0.156	0.339	
		2013	1.510	0.131	0.312	
		2014	-1.227	0.220	0.422	
		2015	2.627	0.009	0.048	*
		2016	1.437	0.151	0.334	
		2017	-2.051	0.040	0.139	
		2018	-1.169	0.242	0.445	
mayavi	comment:PR × member	2013	-1.021	0.307	0.510	
		2014	-2.466	0.014	0.068	.
		2015	-2.357	0.018	0.081	.
		2016	-2.317	0.020	0.085	.
		2017	-0.602	0.547	0.759	
		2018	-1.727	0.084	0.237	
mayavi	comment:PR × nonmember	2011	0.692	0.489	0.707	
		2012	-0.214	0.831	0.925	
		2013	1.112	0.266	0.465	
		2014	-2.745	0.006	0.041	*
		2015	-0.324	0.746	0.886	
		2016	0.454	0.650	0.829	
		2017	-0.353	0.724	0.880	
		2018	0.702	0.483	0.705	
		2012	1.027	0.305	0.508	
		2013	-1.110	0.267	0.465	
		2014	-1.937	0.053	0.168	
mayavi	comment:issue × member					

Continued on next page

Project	Contrast	Year	t-stat	p-val.	p-val adj.	sig.
		2015	-0.429	0.668	0.844	
		2016	-0.424	0.672	0.844	
		2017	-2.689	0.007	0.045	*
		2018	-3.807	0.000	0.002	**
mayavi	comment:issue × nonmember	2011	-2.576	0.010	0.052	.
		2012	-2.591	0.010	0.052	.
		2013	-1.004	0.315	0.517	
		2014	0.275	0.783	0.895	
		2015	-2.269	0.023	0.091	.
		2016	-0.533	0.594	0.782	
		2017	-2.559	0.010	0.054	.
		2018	-0.174	0.862	0.934	
mayavi	post:PR × member	2013	-0.680	0.497	0.714	
		2014	-2.146	0.032	0.120	
		2015	-0.232	0.817	0.918	
		2016	-0.454	0.650	0.829	
		2017	-1.760	0.078	0.228	
		2018	-2.604	0.009	0.051	.
mayavi	post:PR × nonmember	2011	-1.306	0.192	0.380	
		2012	-1.419	0.156	0.339	
		2013	-1.172	0.241	0.445	
		2014	-0.746	0.455	0.671	
		2015	0.522	0.602	0.789	
		2016	0.670	0.503	0.721	
		2017	-1.579	0.114	0.288	
		2018	-1.051	0.293	0.492	
mayavi	post:issue × member	2013	-0.017	0.986	0.992	

Continued on next page

Project	Contrast	Year	t-stat	p-val.	p-val adj.	sig.
		2015	-0.756	0.450	0.668	
		2016	-1.354	0.176	0.364	
		2017	1.489	0.137	0.316	
		2018	-2.442	0.015	0.069	.
mayavi	post:issue × nonmember	2011	-0.660	0.509	0.723	
		2012	-0.542	0.588	0.782	
		2013	2.327	0.020	0.084	.
		2014	0.540	0.590	0.782	
		2015	0.517	0.605	0.790	
		2016	-1.944	0.052	0.168	
		2017	-1.292	0.196	0.386	
		2018	-0.328	0.743	0.886	
numpy	comment:PR × member	2010	0.534	0.594	0.782	
		2011	-1.890	0.059	0.183	
		2012	-6.185	0.000	0.000	***
		2013	0.750	0.453	0.670	
		2014	-0.106	0.916	0.959	
		2015	1.631	0.103	0.270	
		2016	1.719	0.086	0.240	
		2017	1.096	0.273	0.471	
		2018	0.534	0.593	0.782	
numpy	comment:PR × nonmember	2010	-2.405	0.016	0.073	.
		2011	0.406	0.685	0.851	
		2012	-1.495	0.135	0.314	
		2013	-1.458	0.145	0.328	
		2014	-0.782	0.434	0.650	
		2015	-0.281	0.779	0.895	

Continued on next page

Project	Contrast	Year	t-stat	p-val.	p-val adj.	sig.
		2016	1.413	0.158	0.340	
		2017	-0.464	0.643	0.825	
		2018	-0.353	0.724	0.880	
numpy	comment:issue × member	2012	-4.460	0.000	0.000	***
		2013	-3.057	0.002	0.019	*
		2014	-3.937	0.000	0.001	**
		2015	0.840	0.401	0.621	
		2016	-2.341	0.019	0.083	.
		2017	-7.722	0.000	0.000	***
		2018	-3.469	0.001	0.006	**
numpy	comment:issue × nonmember	2012	-2.923	0.003	0.027	*
		2013	-2.628	0.009	0.048	*
		2014	-2.446	0.014	0.069	.
		2015	-2.438	0.015	0.069	.
		2016	-0.876	0.381	0.603	
		2017	-1.366	0.172	0.363	
		2018	-3.461	0.001	0.006	**
numpy	post:PR × member	2010	2.574	0.010	0.052	.
		2011	-0.183	0.854	0.934	
		2012	-3.401	0.001	0.007	**
		2013	-1.886	0.059	0.184	
		2014	-0.784	0.433	0.649	
		2015	-0.179	0.858	0.934	
		2016	-1.528	0.127	0.308	
		2017	-1.084	0.278	0.472	
		2018	-0.343	0.732	0.882	
numpy	post:PR × nonmember	2010	0.121	0.903	0.959	

Continued on next page

Project	Contrast	Year	t-stat	p-val.	p-val adj.	sig.
		2011	-0.282	0.778	0.895	
		2012	-2.133	0.033	0.123	
		2013	0.007	0.994	0.998	
		2014	-1.619	0.105	0.273	
		2015	-1.945	0.052	0.168	
		2016	-1.077	0.282	0.474	
		2017	-2.804	0.005	0.036	*
		2018	-2.678	0.007	0.045	*
numpy	post:issue × member	2012	-3.269	0.001	0.011	*
		2013	-0.661	0.508	0.723	
		2014	-1.714	0.087	0.241	
		2015	-1.084	0.278	0.472	
		2016	-3.548	0.000	0.005	**
		2017	-2.687	0.007	0.045	*
		2018	-1.486	0.137	0.316	
numpy	post:issue × nonmember	2012	-1.510	0.131	0.312	
		2013	-2.802	0.005	0.036	*
		2014	-1.695	0.090	0.244	
		2015	-1.959	0.050	0.166	
		2016	-3.559	0.000	0.005	**
		2017	-2.059	0.039	0.137	
		2018	-3.882	0.000	0.002	**
pandas	comment:PR × member	2011	1.590	0.112	0.284	
		2012	-0.193	0.847	0.934	
		2013	-8.065	0.000	0.000	***
		2014	-1.612	0.107	0.276	
		2015	1.004	0.315	0.517	

Continued on next page

Project	Contrast	Year	t-stat	p-val.	p-val adj.	sig.
		2016	-1.500	0.134	0.314	
		2017	-1.081	0.280	0.473	
		2018	-1.146	0.252	0.453	
pandas	comment:PR × nonmember	2011	1.317	0.188	0.374	
		2012	0.325	0.745	0.886	
		2013	-1.115	0.265	0.465	
		2014	-2.263	0.024	0.092	.
		2015	-1.168	0.243	0.445	
		2016	-2.937	0.003	0.027	*
		2017	-1.644	0.100	0.266	
		2018	-7.995	0.000	0.000	***
pandas	comment:issue × member	2010	0.399	0.690	0.851	
		2011	1.776	0.076	0.223	
		2012	-1.867	0.062	0.190	
		2013	0.274	0.784	0.895	
		2014	2.786	0.005	0.037	*
		2015	0.156	0.876	0.941	
		2016	-1.158	0.247	0.449	
		2017	3.265	0.001	0.011	*
		2018	1.970	0.049	0.165	
pandas	comment:issue × nonmember	2010	1.224	0.221	0.422	
		2011	0.828	0.408	0.629	
		2012	2.873	0.004	0.031	*
		2013	5.223	0.000	0.000	***
		2014	4.586	0.000	0.000	***
		2015	1.085	0.278	0.472	
		2016	0.042	0.967	0.983	

Continued on next page

Project	Contrast	Year	t-stat	p-val.	p-val adj.	sig.
		2017	0.158	0.874	0.941	
		2018	-1.502	0.133	0.314	
pandas	post:PR × member	2011	-0.275	0.783	0.895	
		2012	-2.101	0.036	0.130	
		2013	-1.428	0.153	0.338	
		2014	-0.320	0.749	0.886	
		2015	-2.282	0.022	0.089	.
		2016	-2.649	0.008	0.047	*
		2017	-1.336	0.181	0.367	
		2018	-0.998	0.318	0.520	
pandas	post:PR × nonmember	2011	0.593	0.553	0.763	
		2012	-0.103	0.918	0.959	
		2013	-0.709	0.478	0.700	
		2014	-2.064	0.039	0.137	
		2015	-0.547	0.584	0.782	
		2016	-3.019	0.003	0.021	*
		2017	-2.687	0.007	0.045	*
		2018	4.684	0.000	0.000	***
pandas	post:issue × member	2010	0.579	0.563	0.769	
		2011	-2.248	0.025	0.095	.
		2012	0.260	0.795	0.901	
		2013	3.227	0.001	0.012	*
		2014	3.116	0.002	0.017	*
		2015	0.137	0.891	0.949	
		2016	2.697	0.007	0.045	*
		2017	3.436	0.001	0.007	**
		2018	3.916	0.000	0.001	**

Continued on next page

Project	Contrast	Year	t-stat	p-val.	p-val adj.	sig.
pandas	post:issue × nonmember	2010	0.002	0.998	0.999	
		2011	1.199	0.230	0.432	
		2012	2.065	0.039	0.137	
		2013	0.513	0.608	0.791	
		2014	0.651	0.515	0.729	
		2015	-0.585	0.559	0.769	
		2016	4.027	0.000	0.001	***
		2017	3.563	0.000	0.005	**
		2018	3.339	0.001	0.009	**
scikit-image	comment:PR × member	2011	0.263	0.793	0.901	
		2012	1.426	0.154	0.338	
		2013	2.952	0.003	0.026	*
		2014	1.746	0.081	0.232	
		2015	2.706	0.007	0.045	*
		2016	1.760	0.078	0.228	
		2017	2.340	0.019	0.083	.
		2018	0.841	0.401	0.621	
scikit-image	comment:PR × nonmember	2011	1.269	0.205	0.398	
		2012	-1.509	0.131	0.312	
		2013	0.914	0.361	0.581	
		2014	1.709	0.087	0.242	
		2015	-1.380	0.168	0.358	
		2016	1.921	0.055	0.172	
		2017	5.540	0.000	0.000	***
		2018	6.461	0.000	0.000	***
		2011	0.404	0.686	0.851	
		2012	0.224	0.823	0.920	

Continued on next page

scikit-image	comment:issue × member					

Project	Contrast	Year	t-stat	p-val.	p-val adj.	sig.
		2013	-0.108	0.914	0.959	
		2014	-1.250	0.211	0.409	
		2015	2.118	0.034	0.126	
		2016	0.200	0.842	0.934	
		2017	2.379	0.017	0.078	.
		2018	2.491	0.013	0.064	.
scikit-image	comment:issue × nonmember	2011	-0.424	0.672	0.844	
		2012	-0.694	0.488	0.707	
		2013	-0.857	0.392	0.613	
		2014	0.018	0.986	0.992	
		2015	1.125	0.261	0.465	
		2016	-0.445	0.657	0.836	
		2017	0.877	0.381	0.603	
		2018	2.315	0.021	0.085	.
scikit-image	post:PR × member	2011	-1.567	0.117	0.293	
		2012	-1.446	0.148	0.334	
		2013	0.374	0.708	0.867	
		2014	-1.168	0.243	0.445	
		2015	0.089	0.929	0.959	
		2016	0.597	0.551	0.762	
		2017	2.404	0.016	0.073	.
		2018	6.409	0.000	0.000	***
scikit-image	post:PR × nonmember	2011	0.564	0.573	0.781	
		2012	-0.091	0.927	0.959	
		2013	2.327	0.020	0.084	.
		2014	0.753	0.452	0.669	
		2015	1.214	0.225	0.426	

Continued on next page

Project	Contrast	Year	t-stat	p-val.	p-val adj.	sig.
		2016	5.060	0.000	0.000	***
		2017	8.701	0.000	0.000	***
		2018	6.175	0.000	0.000	***
scikit-image	post:issue × member	2011	0.275	0.783	0.895	
		2012	-2.034	0.042	0.144	
		2013	-1.594	0.111	0.283	
		2014	-1.821	0.069	0.206	
		2015	-0.646	0.518	0.732	
		2016	-2.670	0.008	0.045	*
		2017	-1.345	0.178	0.364	
		2018	1.344	0.179	0.364	
scikit-image	post:issue × nonmember	2011	0.187	0.851	0.934	
		2012	-2.148	0.032	0.120	
		2013	-0.619	0.536	0.748	
		2014	0.554	0.580	0.782	
		2015	1.164	0.245	0.446	
		2016	-1.088	0.277	0.472	
		2017	-1.276	0.202	0.396	
		2018	-0.469	0.639	0.823	
scikit-learn	comment:PR × member	2010	-0.540	0.590	0.782	
		2011	2.405	0.016	0.073	.
		2012	4.203	0.000	0.000	***
		2013	5.497	0.000	0.000	***
		2014	3.418	0.001	0.007	**
		2015	-2.091	0.037	0.132	
		2016	-1.540	0.124	0.305	
		2017	0.112	0.911	0.959	

Continued on next page

Project	Contrast	Year	t-stat	p-val.	p-val adj.	sig.
		2018	-0.856	0.392	0.613	
scikit-learn	comment:PR × nonmember	2010	2.447	0.014	0.069	.
		2011	-0.917	0.359	0.581	
		2012	-0.172	0.864	0.934	
		2013	1.794	0.073	0.217	
		2014	1.533	0.125	0.307	
		2015	0.311	0.756	0.889	
		2016	1.121	0.262	0.465	
		2017	-1.654	0.098	0.262	
		2018	3.284	0.001	0.011	*
scikit-learn	comment:issue × member	2010	-0.333	0.739	0.886	
		2011	1.578	0.115	0.288	
		2012	3.985	0.000	0.001	**
		2013	2.317	0.020	0.085	.
		2014	-0.273	0.785	0.895	
		2015	-2.754	0.006	0.040	*
		2016	0.343	0.731	0.882	
		2017	1.951	0.051	0.168	
		2018	-0.370	0.711	0.868	
scikit-learn	comment:issue × nonmember	2010	-1.137	0.256	0.458	
		2011	0.691	0.489	0.707	
		2012	-0.279	0.780	0.895	
		2013	-2.286	0.022	0.089	.
		2014	-1.106	0.269	0.465	
		2015	-0.608	0.543	0.756	
		2016	0.787	0.432	0.649	
		2017	1.404	0.160	0.343	

Continued on next page

Project	Contrast	Year	t-stat	p-val.	p-val adj.	sig.
		2018	2.801	0.005	0.036	*
scikit-learn	post:PR × member	2010	-2.585	0.010	0.052	.
		2011	5.644	0.000	0.000	***
		2012	3.036	0.002	0.021	*
		2013	3.852	0.000	0.002	**
		2014	2.599	0.009	0.051	.
		2015	1.270	0.204	0.398	
		2016	0.620	0.535	0.748	
		2017	-0.349	0.727	0.882	
		2018	-2.462	0.014	0.068	.
scikit-learn	post:PR × nonmember	2010	-0.092	0.926	0.959	
		2011	0.400	0.689	0.851	
		2012	2.667	0.008	0.045	*
		2013	0.894	0.371	0.594	
		2014	1.351	0.177	0.364	
		2015	-0.881	0.379	0.603	
		2016	1.110	0.267	0.465	
		2017	0.175	0.861	0.934	
		2018	-5.046	0.000	0.000	***
scikit-learn	post:issue × member	2010	-0.490	0.624	0.810	
		2011	0.305	0.761	0.891	
		2012	2.072	0.038	0.136	
		2013	-0.322	0.747	0.886	
		2014	-1.367	0.172	0.363	
		2015	-0.950	0.342	0.557	
		2016	0.810	0.418	0.641	
		2017	1.938	0.053	0.168	

Continued on next page

Project	Contrast	Year	t-stat	p-val.	p-val adj.	sig.
		2018	-1.752	0.080	0.231	
scikit-learn	post:issue × nonmember	2010	0.096	0.923	0.959	
		2011	0.274	0.784	0.895	
		2012	-1.935	0.053	0.168	
		2013	0.137	0.891	0.949	
		2014	1.706	0.088	0.242	
		2015	0.550	0.582	0.782	
		2016	-1.639	0.101	0.267	
		2017	-0.429	0.668	0.844	
		2018	2.450	0.014	0.069	.
scipy	comment:PR × member	2011	0.470	0.638	0.823	
		2012	-0.080	0.936	0.960	
		2013	6.321	0.000	0.000	***
		2014	-0.550	0.582	0.782	
		2015	5.066	0.000	0.000	***
		2016	7.714	0.000	0.000	***
		2017	1.327	0.185	0.371	
		2018	2.964	0.003	0.025	*
scipy	comment:PR × nonmember	2011	0.550	0.582	0.782	
		2012	1.331	0.183	0.370	
		2013	-0.019	0.985	0.992	
		2014	0.864	0.387	0.610	
		2015	4.199	0.000	0.000	***
		2016	2.335	0.020	0.084	.
		2017	1.538	0.124	0.305	
		2018	4.730	0.000	0.000	***
scipy	comment:issue × member	2013	-1.298	0.194	0.384	

Continued on next page

Project	Contrast	Year	t-stat	p-val.	p-val adj.	sig.
		2014	1.355	0.175	0.364	
		2015	1.828	0.068	0.205	
		2016	0.546	0.585	0.782	
		2017	-0.313	0.754	0.889	
		2018	-1.964	0.050	0.165	
scipy	comment:issue × nonmember	2013	-0.520	0.603	0.789	
		2014	1.203	0.229	0.431	
		2015	3.083	0.002	0.018	*
		2016	0.078	0.938	0.960	
		2017	1.478	0.139	0.319	
		2018	2.634	0.008	0.048	*
scipy	post:PR × member	2011	-0.108	0.914	0.959	
		2012	0.475	0.635	0.821	
		2013	-0.198	0.843	0.934	
		2014	-0.794	0.427	0.649	
		2015	0.690	0.490	0.707	
		2016	1.212	0.226	0.427	
		2017	-0.804	0.421	0.644	
		2018	-1.365	0.172	0.363	
scipy	post:PR × nonmember	2011	1.017	0.309	0.510	
		2012	-0.375	0.708	0.867	
		2013	-0.001	0.999	0.999	
		2014	0.580	0.562	0.769	
		2015	2.742	0.006	0.041	*
		2016	0.408	0.683	0.851	
		2017	0.760	0.447	0.667	
		2018	-0.561	0.575	0.782	

Continued on next page

Project	Contrast	Year	t-stat	p-val.	p-val adj.	sig.
scipy	post:issue × member	2013	-1.485	0.138	0.316	
		2014	-0.926	0.355	0.575	
		2015	-0.259	0.796	0.901	
		2016	-1.515	0.130	0.312	
		2017	-3.157	0.002	0.015	*
		2018	-1.822	0.069	0.206	
scipy	post:issue × nonmember	2013	0.327	0.744	0.886	
		2014	-0.025	0.980	0.992	
		2015	-1.672	0.095	0.254	
		2016	-0.089	0.929	0.959	
		2017	1.153	0.249	0.449	
		2018	-1.153	0.249	0.449	
sphinx-gallery	comment:PR × member	2015	1.543	0.123	0.305	
		2016	-0.309	0.757	0.889	
		2017	-1.087	0.277	0.472	
		2018	2.218	0.027	0.102	
sphinx-gallery	comment:PR × nonmember	2014	-0.283	0.777	0.895	
		2015	0.273	0.785	0.895	
		2016	-1.107	0.268	0.465	
		2017	-0.230	0.818	0.918	
		2018	0.869	0.385	0.608	
sphinx-gallery	comment:issue × member	2015	-0.334	0.739	0.886	
		2016	1.352	0.176	0.364	
		2017	2.672	0.008	0.045	*
		2018	3.503	0.000	0.006	**
		2014	0.058	0.954	0.972	
		2015	-0.023	0.982	0.992	
sphinx-gallery	comment:issue × nonmember					

Continued on next page

Project	Contrast	Year	t-stat	p-val.	p-val adj.	sig.
		2016	1.222	0.222	0.422	
		2017	0.637	0.524	0.738	
		2018	2.301	0.021	0.087	.
sphinx-gallery	post:PR × member	2015	1.495	0.135	0.314	
		2016	0.139	0.889	0.949	
		2017	-0.223	0.824	0.920	
		2018	0.140	0.889	0.949	
sphinx-gallery	post:PR × nonmember	2014	1.736	0.083	0.235	
		2015	1.437	0.151	0.334	
		2016	-0.792	0.429	0.649	
		2017	1.437	0.151	0.334	
		2018	0.853	0.394	0.614	
sphinx-gallery	post:issue × member	2015	-0.440	0.660	0.838	
		2016	-2.083	0.037	0.134	
		2017	-0.177	0.859	0.934	
		2018	2.010	0.044	0.151	
sphinx-gallery	post:issue × nonmember	2014	1.349	0.177	0.364	
		2015	-0.184	0.854	0.934	
		2016	-0.791	0.429	0.649	
		2017	0.196	0.845	0.934	
		2018	-2.166	0.030	0.116	

Supplementary Table 3

*Results of analyses of changes in sentiment over time by project, activity (posted issue [`post:issue`], comment on an issue [`comment:issue`], posted pull request [`post:PR`], or comment on a pull request [`comment:PR`]), and membership (`member` or `nonmember` at time of post). All p-values adjusted for multiple comparisons using Benjamini-Hochberg. Legend: . = $p < .10$; * = $p < .05$; ** = $p < .001$; *** = $p < .0001$*

Project	Contrast	Year	t-stat	p-val.	p-val adj.	sig.
matplotlib	comment:PR × member	2011	-0.210	0.834	0.931	
		2012	-2.135	0.033	0.127	
		2013	0.784	0.433	0.709	
		2014	-2.363	0.018	0.081	.
		2015	-3.106	0.002	0.014	*
		2016	-2.742	0.006	0.034	*
		2017	-3.346	0.001	0.007	**
		2018	-4.343	0.000	0.000	***
matplotlib	comment:PR × nonmember	2011	-1.296	0.195	0.435	
		2012	2.967	0.003	0.019	*
		2013	-0.261	0.794	0.919	
		2014	-1.121	0.262	0.544	
		2015	-1.475	0.140	0.363	
		2016	-0.194	0.846	0.937	
		2017	0.513	0.608	0.811	
		2018	0.272	0.785	0.918	
matplotlib	comment:issue × member	2011	0.583	0.560	0.792	
		2012	2.052	0.040	0.150	
		2013	3.060	0.002	0.015	*
		2014	2.364	0.018	0.081	.
		2015	3.845	0.000	0.001	**
		2016	3.633	0.000	0.003	**
		2017	3.031	0.002	0.016	*
		2018	4.867	0.000	0.000	***
matplotlib	comment:issue × nonmember	2011	1.023	0.306	0.601	
		2012	-2.552	0.011	0.053	.
		2013	-1.022	0.307	0.601	

Continued on next page

Project	Contrast	Year	t-stat	p-val.	p-val adj.	sig.
		2014	-2.421	0.015	0.071	.
		2015	1.800	0.072	0.229	
		2016	1.246	0.213	0.462	
		2017	1.195	0.232	0.496	
		2018	2.718	0.007	0.036	*
matplotlib	post:PR × member	2011	1.392	0.164	0.400	
		2012	5.482	0.000	0.000	***
		2013	1.664	0.096	0.278	
		2014	2.002	0.045	0.161	
		2015	2.898	0.004	0.023	*
		2016	3.891	0.000	0.001	**
		2017	3.508	0.000	0.004	**
		2018	4.079	0.000	0.001	***
matplotlib	post:PR × nonmember	2011	-0.243	0.808	0.920	
		2012	0.913	0.361	0.645	
		2013	-0.269	0.788	0.918	
		2014	0.188	0.851	0.937	
		2015	-0.974	0.330	0.621	
		2016	0.028	0.978	0.982	
		2017	0.241	0.810	0.920	
		2018	0.240	0.810	0.920	
matplotlib	post:issue × member	2011	6.754	0.000	0.000	***
		2012	0.527	0.598	0.801	
		2013	0.460	0.645	0.826	
		2014	1.036	0.300	0.596	
		2015	2.036	0.042	0.151	
		2016	2.614	0.009	0.046	*

Continued on next page

Project	Contrast	Year	t-stat	p-val.	p-val adj.	sig.
		2017	1.380	0.168	0.404	
		2018	0.976	0.329	0.621	
matplotlib	post:issue × nonmember	2011	-1.633	0.103	0.286	
		2012	-0.173	0.862	0.943	
		2013	1.787	0.074	0.232	
		2014	1.196	0.232	0.496	
		2015	-0.902	0.367	0.645	
		2016	1.741	0.082	0.245	
		2017	-2.072	0.038	0.145	
		2018	-0.998	0.318	0.613	
mayavi	comment:PR × member	2013	0.575	0.566	0.793	
		2014	2.237	0.025	0.105	
		2015	0.765	0.444	0.711	
		2016	4.453	0.000	0.000	***
		2017	5.386	0.000	0.000	***
		2018	3.407	0.001	0.006	**
mayavi	comment:PR × nonmember	2011	1.684	0.092	0.270	
		2012	2.840	0.005	0.027	*
		2013	3.672	0.000	0.003	**
		2014	-0.272	0.786	0.918	
		2015	0.669	0.504	0.750	
		2016	2.454	0.014	0.068	.
		2017	-0.164	0.869	0.943	
		2018	1.791	0.073	0.232	
mayavi	comment:issue × member	2012	-0.619	0.536	0.775	
		2013	-0.726	0.468	0.725	
		2014	-2.992	0.003	0.018	*

Continued on next page

Project	Contrast	Year	t-stat	p-val.	p-val adj.	sig.
		2015	1.745	0.081	0.244	
		2016	3.713	0.000	0.002	**
		2017	-0.977	0.329	0.621	
		2018	-2.429	0.015	0.070	.
mayavi	comment:issue × nonmember	2011	-1.772	0.076	0.238	
		2012	-2.183	0.029	0.116	
		2013	0.132	0.895	0.951	
		2014	-0.228	0.819	0.925	
		2015	-0.925	0.355	0.636	
		2016	2.849	0.004	0.027	*
		2017	1.312	0.189	0.430	
		2018	5.882	0.000	0.000	***
mayavi	post:PR × member	2013	-0.678	0.498	0.750	
		2014	-1.764	0.078	0.239	
		2015	0.371	0.711	0.879	
		2016	1.723	0.085	0.252	
		2017	0.438	0.662	0.833	
		2018	-3.214	0.001	0.010	*
mayavi	post:PR × nonmember	2011	0.168	0.867	0.943	
		2012	1.362	0.173	0.412	
		2013	-0.011	0.991	0.991	
		2014	0.694	0.487	0.743	
		2015	-0.440	0.660	0.833	
		2016	0.759	0.448	0.712	
		2017	0.490	0.624	0.816	
		2018	-0.071	0.943	0.968	
mayavi	post:issue × member	2013	-0.572	0.567	0.793	

Continued on next page

Project	Contrast	Year	t-stat	p-val.	p-val adj.	sig.
		2015	0.959	0.337	0.623	
		2016	1.133	0.257	0.539	
		2017	0.531	0.596	0.801	
		2018	-0.439	0.661	0.833	
mayavi	post:issue × nonmember	2011	1.042	0.297	0.595	
		2012	0.447	0.655	0.833	
		2013	0.804	0.421	0.704	
		2014	2.431	0.015	0.070	.
		2015	3.641	0.000	0.003	**
		2016	2.795	0.005	0.030	*
		2017	2.135	0.033	0.127	
		2018	4.251	0.000	0.000	***
numpy	comment:PR × member	2010	-2.524	0.012	0.057	.
		2011	-5.381	0.000	0.000	***
		2012	-9.248	0.000	0.000	***
		2013	-4.219	0.000	0.000	***
		2014	0.869	0.385	0.662	
		2015	2.704	0.007	0.037	*
		2016	0.615	0.538	0.776	
		2017	-5.809	0.000	0.000	***
		2018	-0.557	0.577	0.796	
numpy	comment:PR × nonmember	2010	0.059	0.953	0.973	
		2011	0.534	0.593	0.801	
		2012	-2.476	0.013	0.065	.
		2013	-2.401	0.016	0.075	.
		2014	-0.777	0.437	0.709	
		2015	1.247	0.212	0.462	

Continued on next page

Project	Contrast	Year	t-stat	p-val.	p-val adj.	sig.
		2016	1.473	0.141	0.363	
		2017	-1.066	0.286	0.578	
		2018	0.658	0.511	0.750	
numpy	comment:issue × member	2012	-4.025	0.000	0.001	***
		2013	-4.751	0.000	0.000	***
		2014	-5.043	0.000	0.000	***
		2015	-1.394	0.163	0.400	
		2016	-3.077	0.002	0.015	*
		2017	-4.283	0.000	0.000	***
		2018	-2.714	0.007	0.036	*
numpy	comment:issue × nonmember	2012	-2.146	0.032	0.125	
		2013	-0.884	0.377	0.655	
		2014	-0.534	0.593	0.801	
		2015	1.309	0.191	0.430	
		2016	-2.200	0.028	0.112	
		2017	0.588	0.556	0.792	
		2018	3.663	0.000	0.003	**
numpy	post:PR × member	2010	0.162	0.871	0.943	
		2011	-0.794	0.427	0.707	
		2012	-2.756	0.006	0.033	*
		2013	-1.954	0.051	0.176	
		2014	-0.261	0.794	0.919	
		2015	-1.517	0.129	0.345	
		2016	-1.356	0.175	0.412	
		2017	-0.666	0.505	0.750	
		2018	-0.631	0.528	0.772	
numpy	post:PR × nonmember	2010	1.358	0.174	0.412	

Continued on next page

Project	Contrast	Year	t-stat	p-val.	p-val adj.	sig.
		2011	-0.799	0.424	0.704	
		2012	-1.119	0.263	0.544	
		2013	-0.771	0.440	0.710	
		2014	-0.963	0.335	0.623	
		2015	-0.947	0.344	0.625	
		2016	-1.105	0.269	0.552	
		2017	-0.105	0.916	0.957	
		2018	0.364	0.716	0.883	
numpy	post:issue × member	2012	0.487	0.626	0.816	
		2013	-0.944	0.345	0.626	
		2014	0.457	0.647	0.827	
		2015	-0.113	0.910	0.956	
		2016	-0.218	0.827	0.931	
		2017	0.253	0.800	0.920	
		2018	1.025	0.305	0.601	
numpy	post:issue × nonmember	2012	-0.757	0.449	0.712	
		2013	0.336	0.737	0.896	
		2014	-2.816	0.005	0.029	*
		2015	-1.456	0.145	0.369	
		2016	-1.315	0.189	0.430	
		2017	0.824	0.410	0.693	
		2018	0.472	0.637	0.819	
pandas	comment:PR × member	2011	8.185	0.000	0.000	***
		2012	7.451	0.000	0.000	***
		2013	-7.231	0.000	0.000	***
		2014	-1.461	0.144	0.368	
		2015	1.862	0.063	0.207	

Continued on next page

Project	Contrast	Year	t-stat	p-val.	p-val adj.	sig.
		2016	-0.156	0.876	0.945	
		2017	5.417	0.000	0.000	***
		2018	11.183	0.000	0.000	***
pandas	comment:PR × nonmember	2011	1.443	0.149	0.377	
		2012	1.759	0.079	0.240	
		2013	0.948	0.343	0.625	
		2014	0.170	0.865	0.943	
		2015	-0.722	0.470	0.726	
		2016	-2.669	0.008	0.040	*
		2017	0.204	0.838	0.931	
		2018	0.504	0.615	0.812	
pandas	comment:issue × member	2010	0.243	0.808	0.920	
		2011	-0.904	0.366	0.645	
		2012	-3.540	0.000	0.004	**
		2013	-0.661	0.509	0.750	
		2014	1.185	0.236	0.501	
		2015	-0.218	0.828	0.931	
		2016	-1.083	0.279	0.567	
		2017	0.612	0.540	0.776	
		2018	2.906	0.004	0.023	*
pandas	comment:issue × nonmember	2010	-0.212	0.832	0.931	
		2011	2.073	0.038	0.145	
		2012	3.458	0.001	0.005	**
		2013	4.576	0.000	0.000	***
		2014	3.234	0.001	0.010	**
		2015	0.533	0.594	0.801	
		2016	1.310	0.190	0.430	

Continued on next page

Project	Contrast	Year	t-stat	p-val.	p-val adj.	sig.
		2017	-1.624	0.104	0.289	
		2018	-5.942	0.000	0.000	***
pandas	post:PR × member	2011	0.672	0.502	0.750	
		2012	-2.687	0.007	0.038	*
		2013	0.049	0.961	0.973	
		2014	-0.092	0.926	0.960	
		2015	-0.659	0.510	0.750	
		2016	-1.393	0.164	0.400	
		2017	1.001	0.317	0.613	
		2018	2.038	0.042	0.151	
pandas	post:PR × nonmember	2011	-0.508	0.612	0.812	
		2012	-1.406	0.160	0.395	
		2013	0.025	0.980	0.982	
		2014	1.479	0.139	0.363	
		2015	0.071	0.944	0.968	
		2016	0.484	0.629	0.816	
		2017	0.118	0.906	0.954	
		2018	-1.250	0.211	0.462	
pandas	post:issue × member	2010	0.880	0.379	0.656	
		2011	-3.969	0.000	0.001	***
		2012	-4.272	0.000	0.000	***
		2013	0.328	0.743	0.900	
		2014	-0.269	0.788	0.918	
		2015	-0.618	0.536	0.775	
		2016	-0.629	0.529	0.772	
		2017	0.741	0.459	0.715	
		2018	1.367	0.172	0.412	

Continued on next page

Project	Contrast	Year	t-stat	p-val.	p-val adj.	sig.
		2010	0.857	0.391	0.666	
		2011	0.318	0.750	0.902	
		2012	-1.018	0.309	0.601	
		2013	-2.332	0.020	0.086	.
pandas	post:issue × nonmember	2014	-1.130	0.259	0.540	
		2015	-2.253	0.024	0.103	
		2016	-3.975	0.000	0.001	***
		2017	-3.664	0.000	0.003	**
		2018	-2.958	0.003	0.020	*
		2011	3.652	0.000	0.003	**
		2012	0.782	0.434	0.709	
		2013	1.465	0.143	0.367	
		2014	-0.538	0.590	0.801	
scikit-image	comment:PR × member	2015	3.072	0.002	0.015	*
		2016	0.303	0.762	0.908	
		2017	1.337	0.181	0.423	
		2018	-5.431	0.000	0.000	***
		2011	0.475	0.635	0.819	
		2012	-2.376	0.018	0.079	.
		2013	-0.753	0.451	0.712	
		2014	-0.398	0.690	0.863	
scikit-image	comment:PR × nonmember	2015	-0.481	0.630	0.816	
		2016	1.564	0.118	0.319	
		2017	-0.106	0.916	0.957	
		2018	0.679	0.497	0.750	
		2011	-1.163	0.245	0.517	
		2012	0.782	0.434	0.709	

Continued on next page

scikit-image	comment:issue × member					

Project	Contrast	Year	t-stat	p-val.	p-val adj.	sig.
		2013	-1.764	0.078	0.239	
		2014	-1.837	0.066	0.216	
		2015	0.099	0.921	0.957	
		2016	-0.565	0.572	0.796	
		2017	-1.039	0.299	0.596	
		2018	-1.303	0.193	0.432	
scikit-image	comment:issue × nonmember	2011	-1.818	0.069	0.224	
		2012	-0.747	0.455	0.715	
		2013	-1.208	0.227	0.489	
		2014	-0.117	0.907	0.954	
		2015	-2.146	0.032	0.125	
		2016	0.254	0.799	0.920	
		2017	-0.484	0.628	0.816	
		2018	2.233	0.026	0.105	
scikit-image	post:PR × member	2011	-0.625	0.532	0.774	
		2012	-3.137	0.002	0.013	*
		2013	-1.642	0.101	0.284	
		2014	-0.930	0.352	0.634	
		2015	-0.546	0.585	0.801	
		2016	-1.710	0.087	0.257	
		2017	-1.940	0.052	0.178	
		2018	-0.959	0.337	0.623	
scikit-image	post:PR × nonmember	2011	-0.129	0.897	0.951	
		2012	-0.035	0.972	0.980	
		2013	0.240	0.810	0.920	
		2014	0.462	0.644	0.826	
		2015	0.661	0.509	0.750	

Continued on next page

Project	Contrast	Year	t-stat	p-val.	p-val adj.	sig.
		2016	-0.084	0.933	0.965	
		2017	-0.878	0.380	0.656	
		2018	0.193	0.847	0.937	
scikit-image	post:issue × member	2011	-0.137	0.891	0.948	
		2012	-0.746	0.456	0.715	
		2013	-1.321	0.186	0.429	
		2014	-1.807	0.071	0.227	
		2015	-2.056	0.040	0.149	
		2016	-1.620	0.105	0.291	
		2017	-1.935	0.053	0.179	
		2018	-0.526	0.599	0.801	
scikit-image	post:issue × nonmember	2011	0.801	0.423	0.704	
		2012	-0.350	0.726	0.888	
		2013	0.717	0.474	0.729	
		2014	1.510	0.131	0.347	
		2015	1.093	0.274	0.560	
		2016	0.147	0.883	0.948	
		2017	0.580	0.562	0.793	
		2018	0.305	0.761	0.908	
scikit-learn	comment:PR × member	2010	1.983	0.047	0.167	
		2011	-0.505	0.613	0.812	
		2012	3.237	0.001	0.010	**
		2013	4.758	0.000	0.000	***
		2014	1.075	0.282	0.572	
		2015	-3.047	0.002	0.016	*
		2016	-1.981	0.048	0.167	
		2017	-1.417	0.157	0.389	

Continued on next page

Project	Contrast	Year	t-stat	p-val.	p-val adj.	sig.
		2018	-11.181	0.000	0.000	***
		2010	-0.311	0.756	0.905	
		2011	0.532	0.595	0.801	
		2012	0.139	0.890	0.948	
		2013	1.926	0.054	0.181	
scikit-learn	comment:PR × nonmember	2014	-0.702	0.483	0.740	
		2015	0.049	0.961	0.973	
		2016	-0.355	0.722	0.887	
		2017	-1.388	0.165	0.400	
		2018	-4.486	0.000	0.000	***
		2010	-0.190	0.849	0.937	
		2011	1.880	0.060	0.200	
		2012	4.909	0.000	0.000	***
		2013	3.104	0.002	0.014	*
scikit-learn	comment:issue × member	2014	1.811	0.070	0.226	
		2015	-0.979	0.328	0.621	
		2016	-0.742	0.458	0.715	
		2017	-0.349	0.727	0.888	
		2018	-5.589	0.000	0.000	***
		2010	0.282	0.778	0.917	
		2011	-1.048	0.295	0.592	
		2012	1.725	0.085	0.252	
		2013	-1.423	0.155	0.389	
scikit-learn	comment:issue × nonmember	2014	-1.653	0.098	0.282	
		2015	-2.440	0.015	0.070	.
		2016	-3.250	0.001	0.010	**
		2017	-2.232	0.026	0.105	

Continued on next page

Project	Contrast	Year	t-stat	p-val.	p-val adj.	sig.
		2018	-4.177	0.000	0.000	***
		2010	-0.384	0.701	0.871	
		2011	-0.538	0.591	0.801	
		2012	0.602	0.547	0.784	
		2013	0.031	0.975	0.982	
scikit-learn	post:PR × member	2014	-0.954	0.340	0.625	
		2015	-0.432	0.666	0.837	
		2016	-1.592	0.111	0.304	
		2017	-3.092	0.002	0.014	*
		2018	-4.746	0.000	0.000	***
		2010	-1.527	0.127	0.342	
		2011	-0.482	0.630	0.816	
		2012	0.776	0.438	0.709	
		2013	1.253	0.210	0.462	
scikit-learn	post:PR × nonmember	2014	-0.897	0.370	0.645	
		2015	0.381	0.703	0.872	
		2016	-0.666	0.505	0.750	
		2017	-0.326	0.744	0.900	
		2018	-0.316	0.752	0.902	
		2010	-0.808	0.419	0.704	
		2011	-0.977	0.329	0.621	
		2012	1.489	0.137	0.358	
		2013	0.139	0.890	0.948	
scikit-learn	post:issue × member	2014	-0.100	0.921	0.957	
		2015	-0.264	0.792	0.919	
		2016	-0.950	0.342	0.625	
		2017	-1.632	0.103	0.286	

Continued on next page

Project	Contrast	Year	t-stat	p-val.	p-val adj.	sig.
		2018	-2.810	0.005	0.029	*
scikit-learn	post:issue × nonmember	2010	-0.774	0.439	0.709	
		2011	0.676	0.499	0.750	
		2012	2.465	0.014	0.066	.
		2013	0.851	0.395	0.669	
		2014	1.329	0.184	0.426	
		2015	3.691	0.000	0.002	**
		2016	1.610	0.107	0.295	
		2017	2.042	0.041	0.151	
		2018	1.156	0.248	0.521	
scipy	comment:PR × member	2011	1.646	0.100	0.283	
		2012	0.970	0.332	0.623	
		2013	8.729	0.000	0.000	***
		2014	3.370	0.001	0.007	**
		2015	3.172	0.002	0.012	*
		2016	5.637	0.000	0.000	***
		2017	4.499	0.000	0.000	***
		2018	7.312	0.000	0.000	***
scipy	comment:PR × nonmember	2011	-0.561	0.575	0.796	
		2012	-0.687	0.492	0.747	
		2013	-0.390	0.696	0.868	
		2014	3.155	0.002	0.012	*
		2015	2.228	0.026	0.105	
		2016	1.017	0.309	0.601	
		2017	2.258	0.024	0.103	
		2018	3.027	0.002	0.016	*
scipy	comment:issue × member	2013	-0.402	0.688	0.862	

Continued on next page

Project	Contrast	Year	t-stat	p-val.	p-val adj.	sig.
		2014	0.863	0.388	0.663	
		2015	-0.765	0.444	0.711	
		2016	1.841	0.066	0.216	
		2017	1.294	0.196	0.435	
		2018	1.650	0.099	0.282	
scipy	comment:issue × nonmember	2013	-2.572	0.010	0.051	.
		2014	0.976	0.329	0.621	
		2015	1.321	0.187	0.429	
		2016	1.941	0.052	0.178	
		2017	1.948	0.051	0.177	
		2018	2.044	0.041	0.151	
scipy	post:PR × member	2011	-0.445	0.656	0.833	
		2012	0.319	0.749	0.902	
		2013	1.521	0.128	0.344	
		2014	0.904	0.366	0.645	
		2015	0.100	0.920	0.957	
		2016	1.669	0.095	0.277	
		2017	-0.488	0.626	0.816	
		2018	-0.556	0.578	0.796	
scipy	post:PR × nonmember	2011	2.348	0.019	0.083	.
		2012	0.337	0.736	0.896	
		2013	-0.355	0.723	0.887	
		2014	-0.292	0.771	0.913	
		2015	0.725	0.468	0.725	
		2016	0.935	0.350	0.631	
		2017	-0.064	0.949	0.971	
		2018	1.289	0.197	0.436	

Continued on next page

Project	Contrast	Year	t-stat	p-val.	p-val adj.	sig.
scipy	post:issue × member	2013	0.502	0.616	0.812	
		2014	0.905	0.365	0.645	
		2015	0.803	0.422	0.704	
		2016	1.117	0.264	0.544	
		2017	0.300	0.764	0.908	
		2018	0.051	0.959	0.973	
scipy	post:issue × nonmember	2013	-0.205	0.838	0.931	
		2014	0.555	0.579	0.796	
		2015	-0.153	0.879	0.946	
		2016	1.418	0.156	0.389	
		2017	2.262	0.024	0.102	
		2018	1.357	0.175	0.412	
sphinx-gallery	comment:PR × member	2015	-2.683	0.007	0.039	*
		2016	-1.493	0.135	0.357	
		2017	-3.609	0.000	0.003	**
		2018	-0.757	0.449	0.712	
sphinx-gallery	comment:PR × nonmember	2014	-0.698	0.485	0.741	
		2015	-2.797	0.005	0.030	*
		2016	-1.755	0.079	0.241	
		2017	-0.585	0.559	0.792	
		2018	-0.072	0.943	0.968	
sphinx-gallery	comment:issue × member	2015	-0.866	0.387	0.663	
		2016	-0.574	0.566	0.793	
		2017	-0.788	0.430	0.709	
		2018	0.558	0.577	0.796	
sphinx-gallery	comment:issue × nonmember	2014	-0.897	0.370	0.645	
		2015	-2.105	0.035	0.135	

Continued on next page

Project	Contrast	Year	t-stat	p-val.	p-val adj.	sig.
		2016	0.236	0.813	0.921	
		2017	2.238	0.025	0.105	
		2018	3.238	0.001	0.010	**
sphinx-gallery	post:PR × member	2015	0.182	0.856	0.940	
		2016	0.141	0.888	0.948	
		2017	0.074	0.941	0.968	
		2018	-0.121	0.904	0.954	
sphinx-gallery	post:PR × nonmember	2014	0.173	0.863	0.943	
		2015	0.966	0.334	0.623	
		2016	-0.244	0.807	0.920	
		2017	1.231	0.218	0.472	
		2018	-0.049	0.961	0.973	
sphinx-gallery	post:issue × member	2015	0.204	0.838	0.931	
		2016	-0.284	0.776	0.917	
		2017	0.589	0.556	0.792	
		2018	0.161	0.872	0.943	
sphinx-gallery	post:issue × nonmember	2014	-0.036	0.971	0.980	
		2015	-1.336	0.182	0.423	
		2016	2.017	0.044	0.157	
		2017	2.655	0.008	0.041	*
		2018	0.277	0.782	0.918	

Supplementary Table 4

*Results of analyses of changes in log of gratitude count over time by project, activity (posted issue [`post:issue`], comment on an issue [`comment:issue`], posted pull request [`post:PR`], or comment on a pull request [`comment:PR`]), and membership (`member` or `nonmember` at time of post). All p-values adjusted for multiple comparisons using Benjamini-Hochberg. Legend: . = $p < .10$; * = $p < .05$; ** = $p < .001$; *** = $p < .0001$*

			t-stat	p-val.	p-val adj.	sig.
1W	$\mu_{\text{PR}} = \mu_{\text{issue}}$		5.542	0.000	0.000	***
	Time opened		-0.975	0.330	0.369	
	Cumulative grateful words on comments		6.722	0.000	0.000	***
	Maximum negative sentiment score		1.367	0.172	0.217	
	Number of comments		9.338	0.000	0.000	***
	Comment member ratio		-8.344	0.000	0.000	***
	Mean sentiment score		6.342	0.000	0.000	***
2W	Time opened	Issue	3.596	0.000	0.001	***
		PR	-2.903	0.004	0.005	**
	Cumulative grateful words on comments	Issue	-1.276	0.202	0.240	
		PR	3.872	0.000	0.000	***
	Maximum negative sentiment score	Issue	4.012	0.000	0.000	***
		PR	6.535	0.000	0.000	***
	Number of comments	Issue	-4.281	0.000	0.000	***
		PR	-8.937	0.000	0.000	***
	Comment member ratio	Issue	4.387	0.000	0.000	***
		PR	-0.824	0.410	0.433	
	Mean sentiment score	Issue	0.059	0.953	0.953	
		PR	3.011	0.003	0.004	**

Supplementary Table 5

*Results of analyses predicting newcomer retention by contribution type and metrics of the community's response to the newcomer. All p-values adjusted for multiple comparisons using Benjamini-Hochberg. Legend: . = $p < .10$; * = $p < .05$; ** = $p < .001$; *** = $p < .0001$*

			t-stat	p-val.	p-val adj.	sig.
1W	$\mu_{\text{PR}} = \mu_{\text{issue}}$		5.531	0.000	0.000	***
	Time opened		-1.296	0.195	0.253	
	Cumulative grateful words on comments		6.701	0.000	0.000	***
	Maximum negative sentiment score		1.400	0.162	0.225	
	Maximum positive sentiment score		9.350	0.000	0.000	***
	Number of comments		-8.363	0.000	0.000	***
	Comment member ratio		6.341	0.000	0.000	***
	Mean sentiment score		3.298	0.001	0.002	**
	Variance sentiment score		-1.120	0.263	0.313	
2W	Time opened	Issue	-2.956	0.003	0.005	**
		PR	-1.275	0.202	0.253	
	Cumulative grateful words on comments	Issue	4.107	0.000	0.000	***
		PR	4.011	0.000	0.000	***
	Maximum negative sentiment score	Issue	6.617	0.000	0.000	***
		PR	-4.288	0.000	0.000	***
	Maximum positive sentiment score	Issue	-9.562	0.000	0.000	***
		PR	4.387	0.000	0.000	***
	Number of comments	Issue	-0.688	0.492	0.534	
		PR	0.060	0.952	0.952	
	Comment member ratio	Issue	3.400	0.001	0.001	**
		PR	10.871	0.000	0.000	***
	Mean sentiment score	Issue	5.300	0.000	0.000	***
		PR	0.452	0.651	0.678	
	Variance sentiment score	Issue	-0.970	0.332	0.377	
		PR	1.527	0.127	0.186	

Continued on next page

	t-stat	p-val.	p-val adj.	sig.

Supplementary Table 6

*Results of analyses predicting newcomer retention by contribution type and metrics of the community's response to the newcomer, with two post hoc additions. All p-values adjusted for multiple comparisons using Benjamini-Hochberg. Legend: . = $p < .10$; * = $p < .05$; ** = $p < .001$; *** = $p < .0001$*

	Issues	Pull requests	Comments on issues	Comments on PRs	Authors	Dependent
matplotlib	4819	7385	28904	36688		14527
mayavi	445	290	1392	758	446	59
numpy	6239	5607	29940	31168	3625	35175
pandas	13412	10264	60673	68656	6806	19806
scikit-image	1195	2104	6397	15182	1023	1756
scikit-learn	5427	6856	34201	68224	5317	5942
scipy	4989	4349	22151	26711	3062	14945
sphinx-gallery	195	223	975	1833	90	236
all	31902	29693	155729	212532	16968	

Supplementary Table 7

*Summary statistics for the current dataset, broken down by project. Dependent package and repository statistics obtained from GitHub's dependency graph in June 2021. Impact percentile from Depsy's 2016 analysis of Python ecosystem (**?**); - `sphinx-gallery` not included in Depsy analysis.*

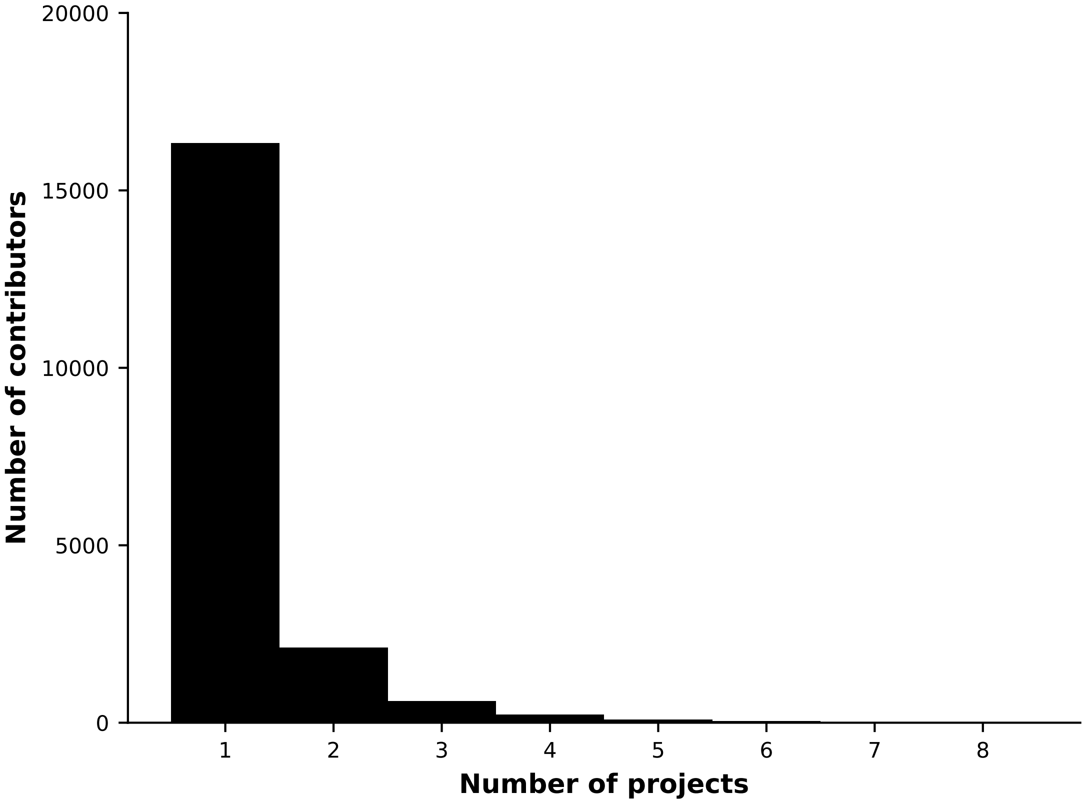

Supplementary Figure 1. Histogram of number of projects to which a single user contributes.
